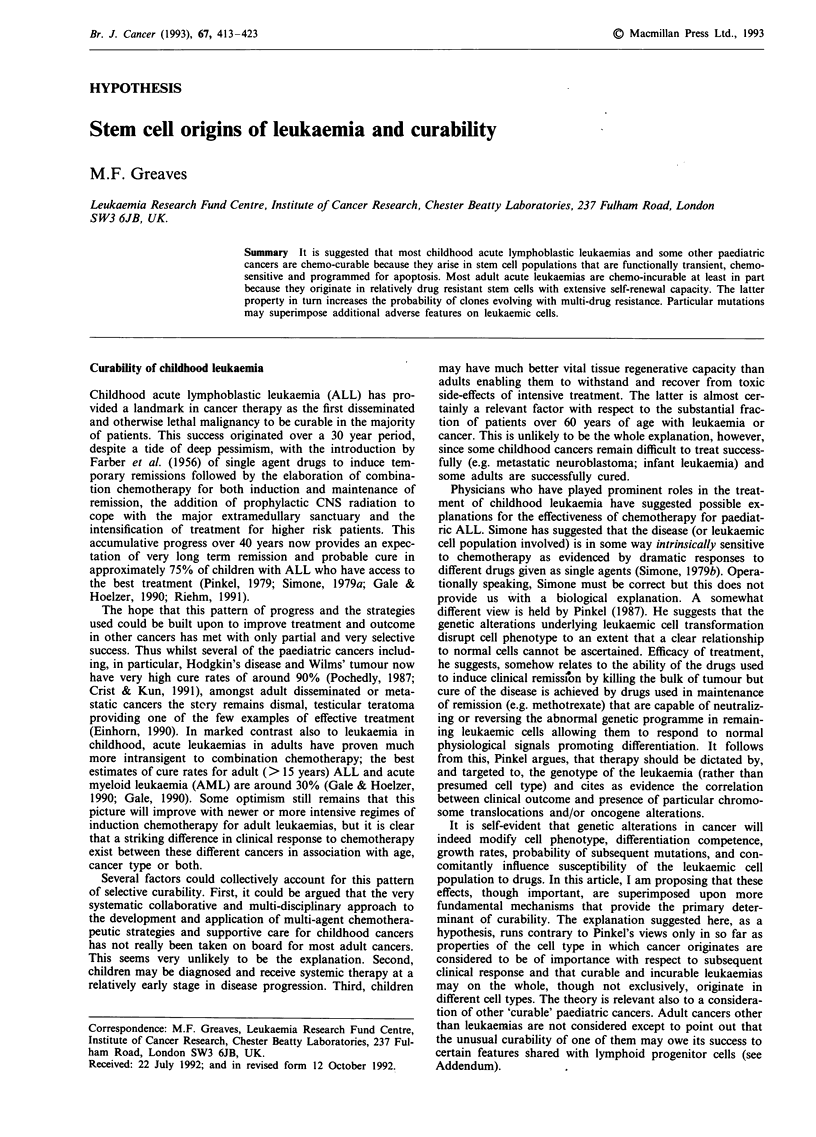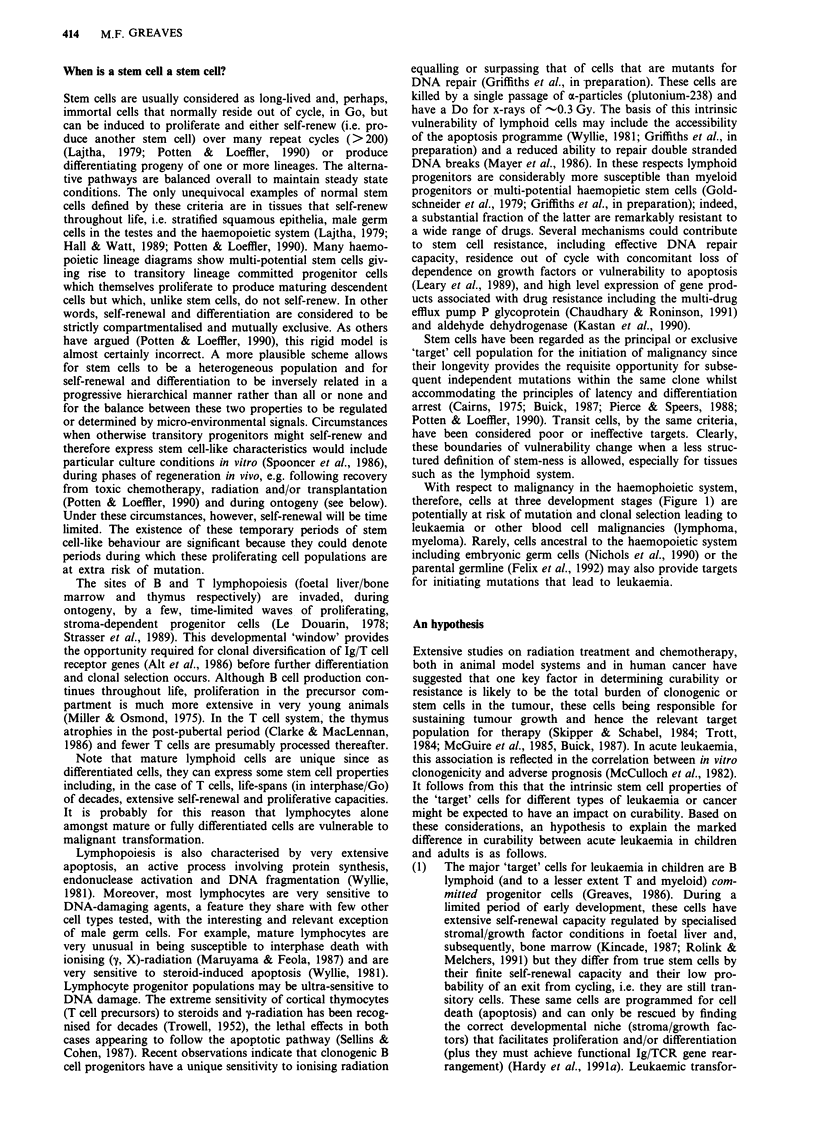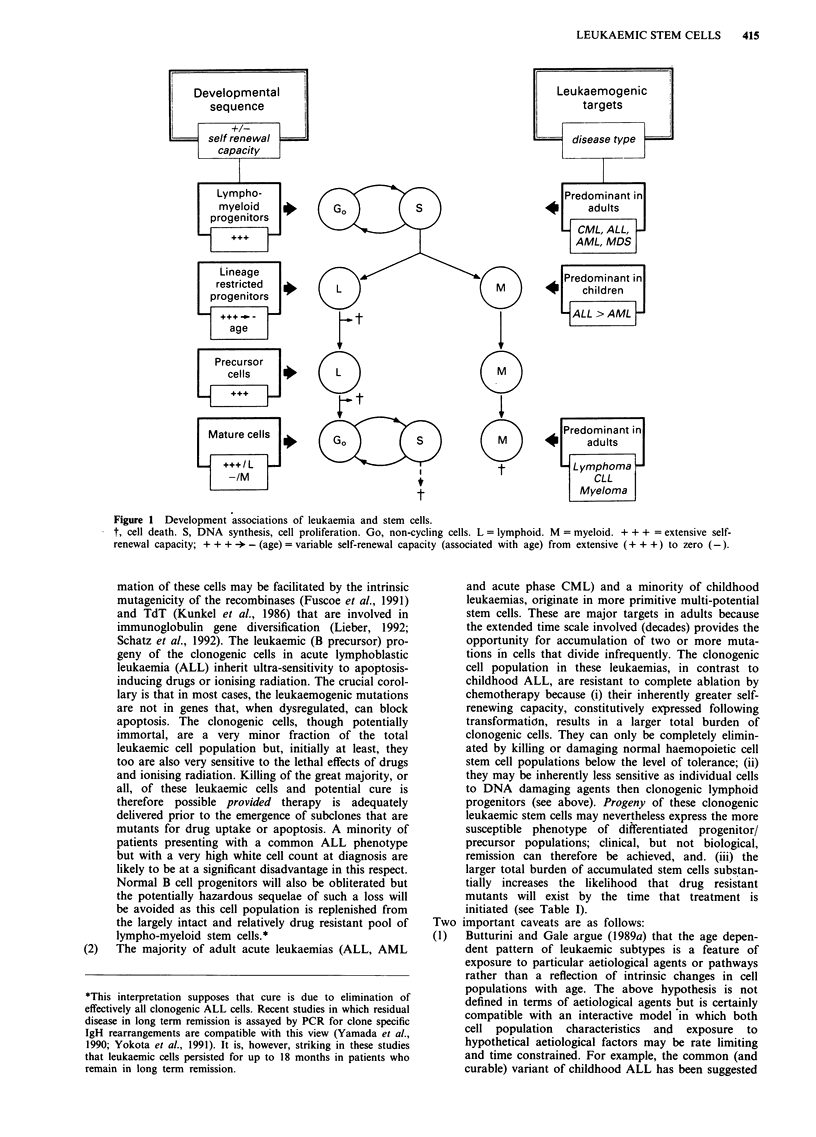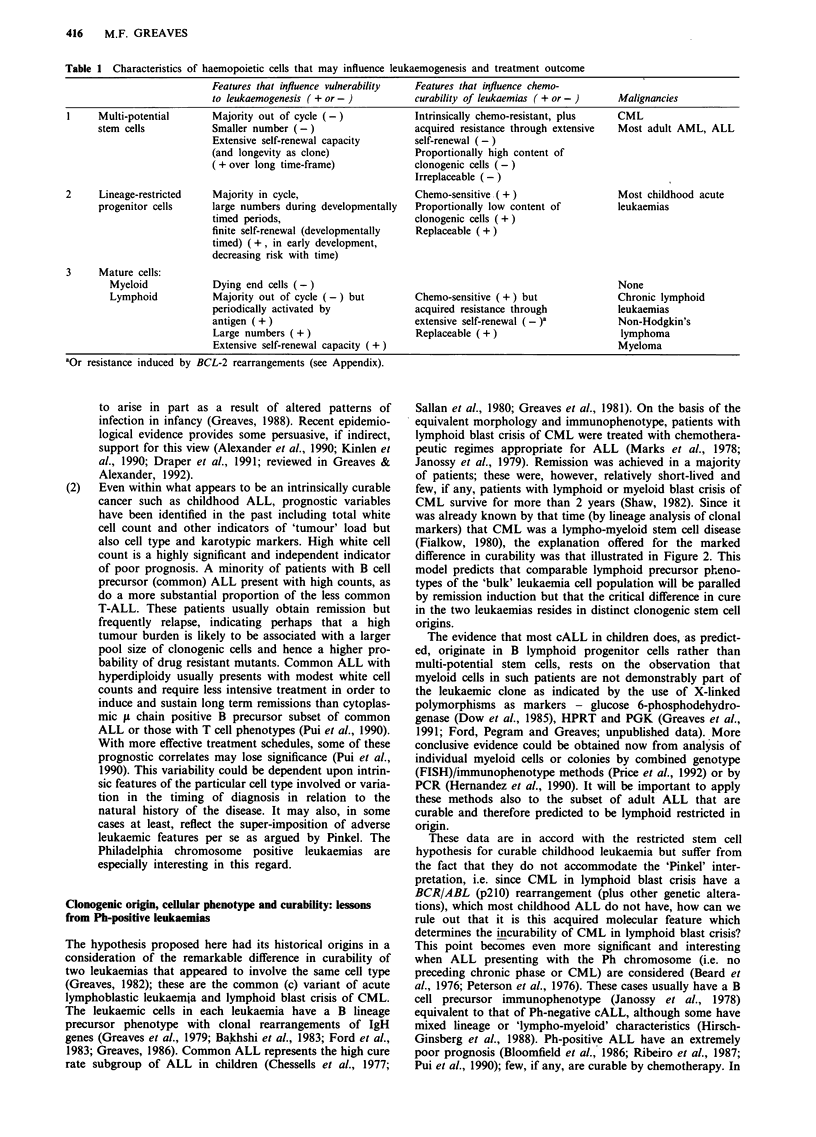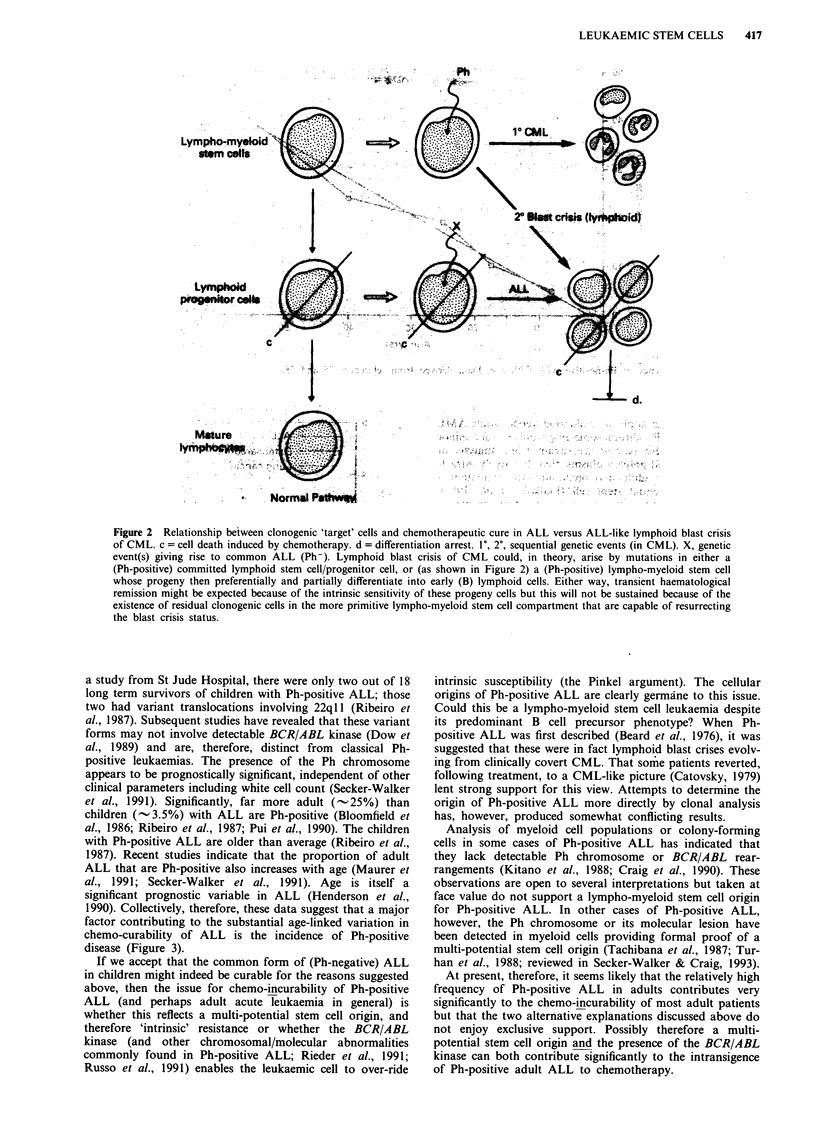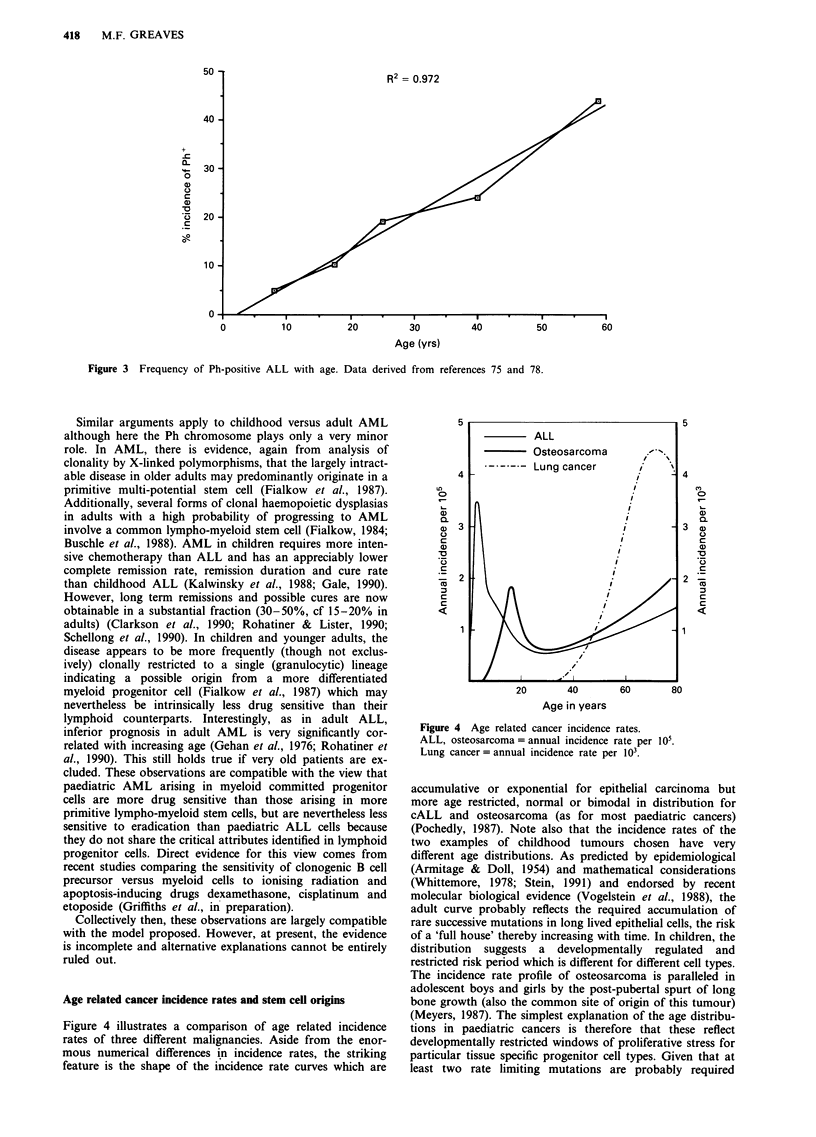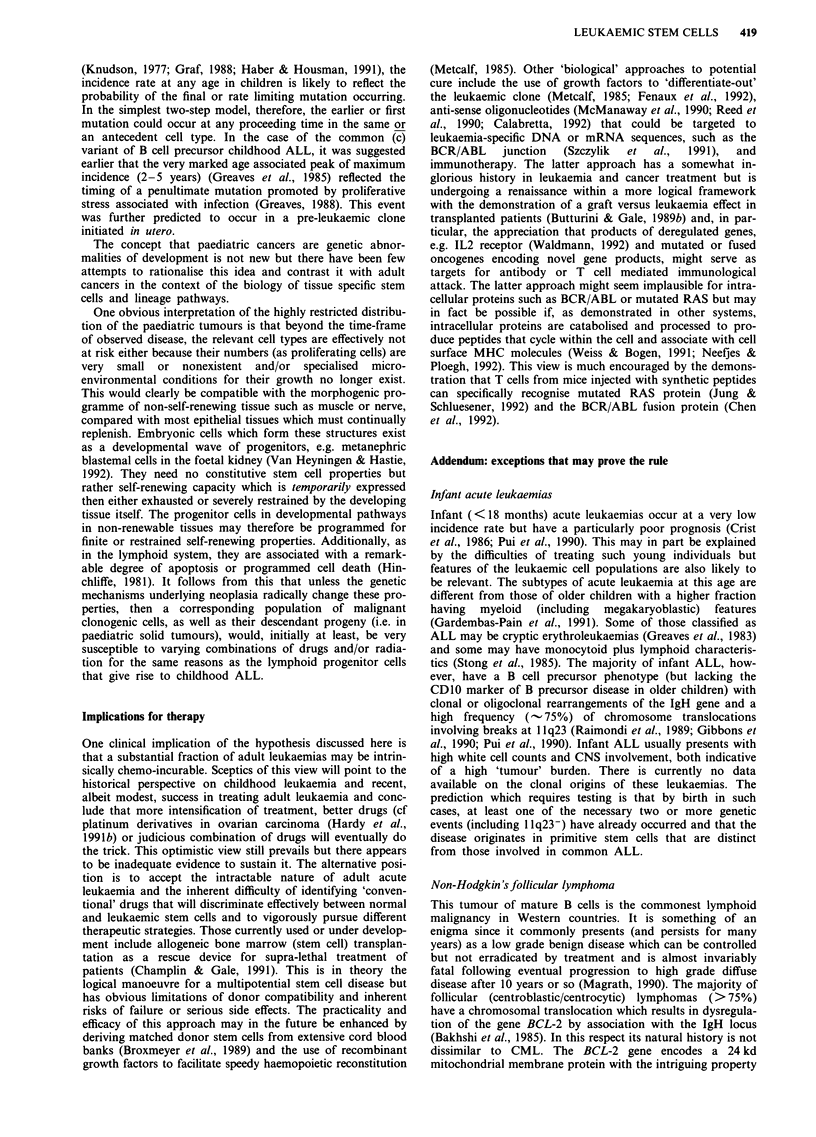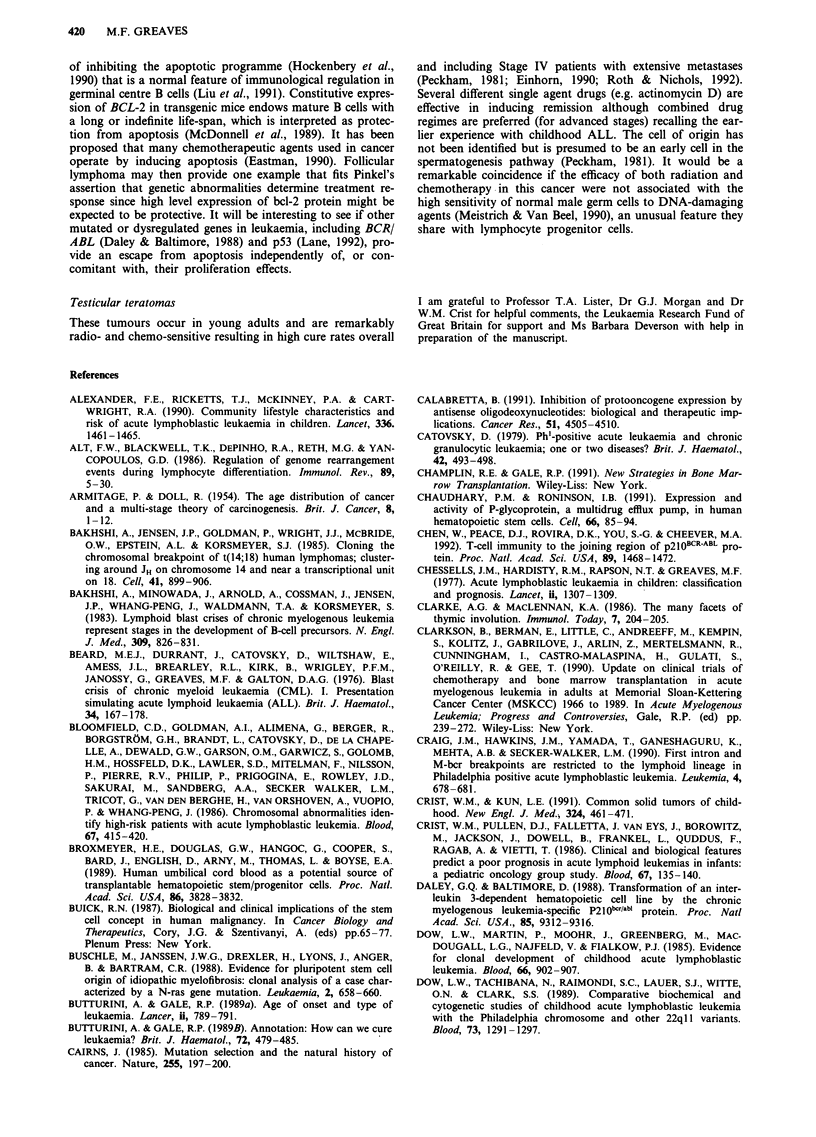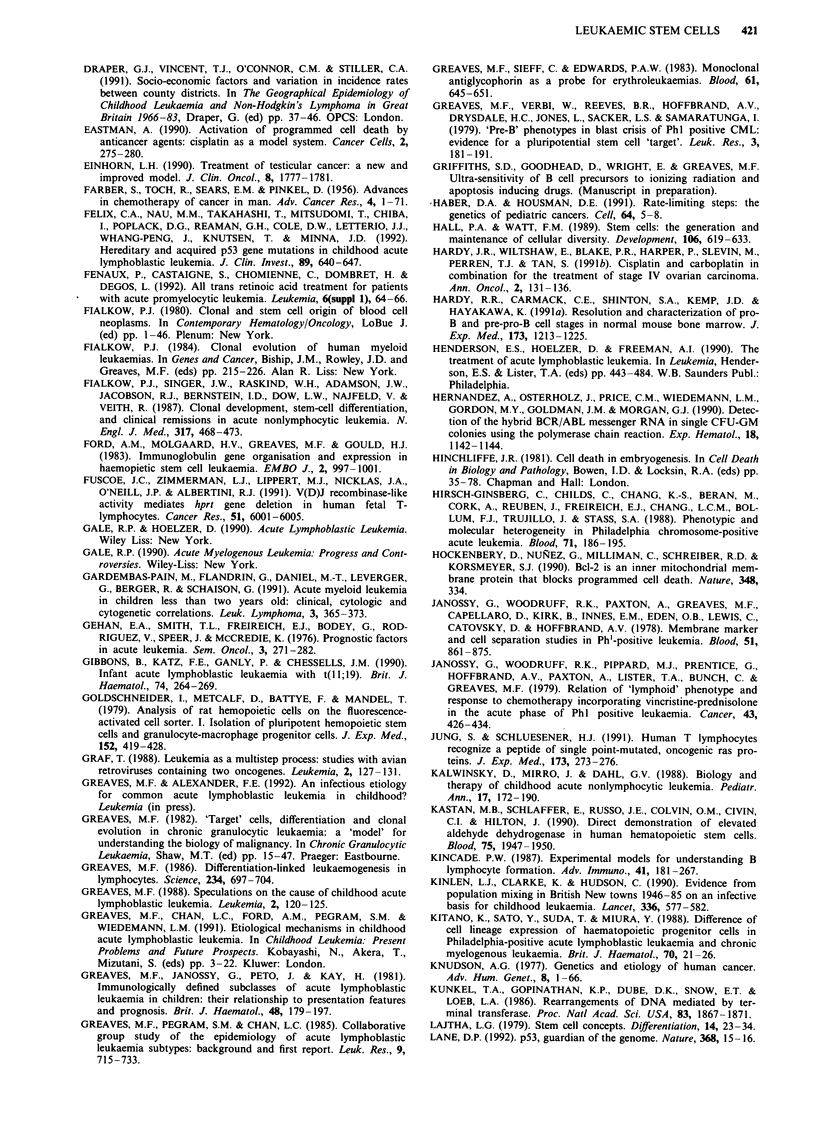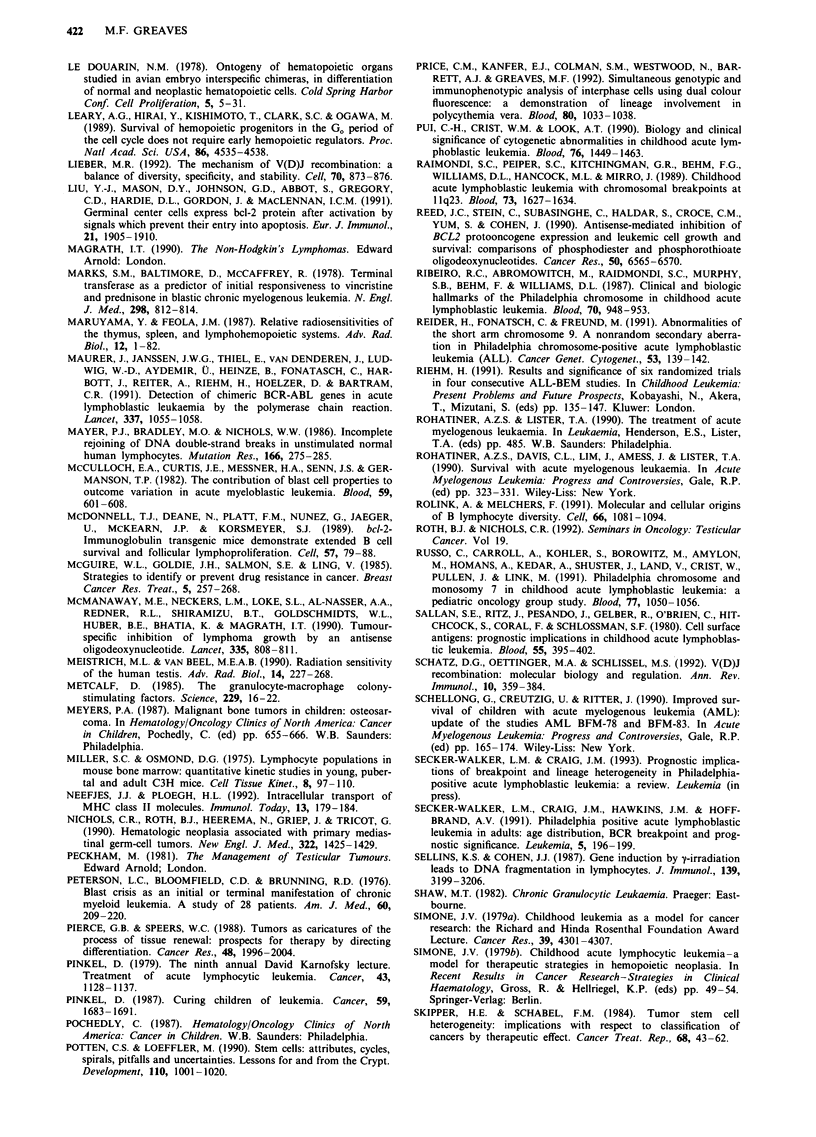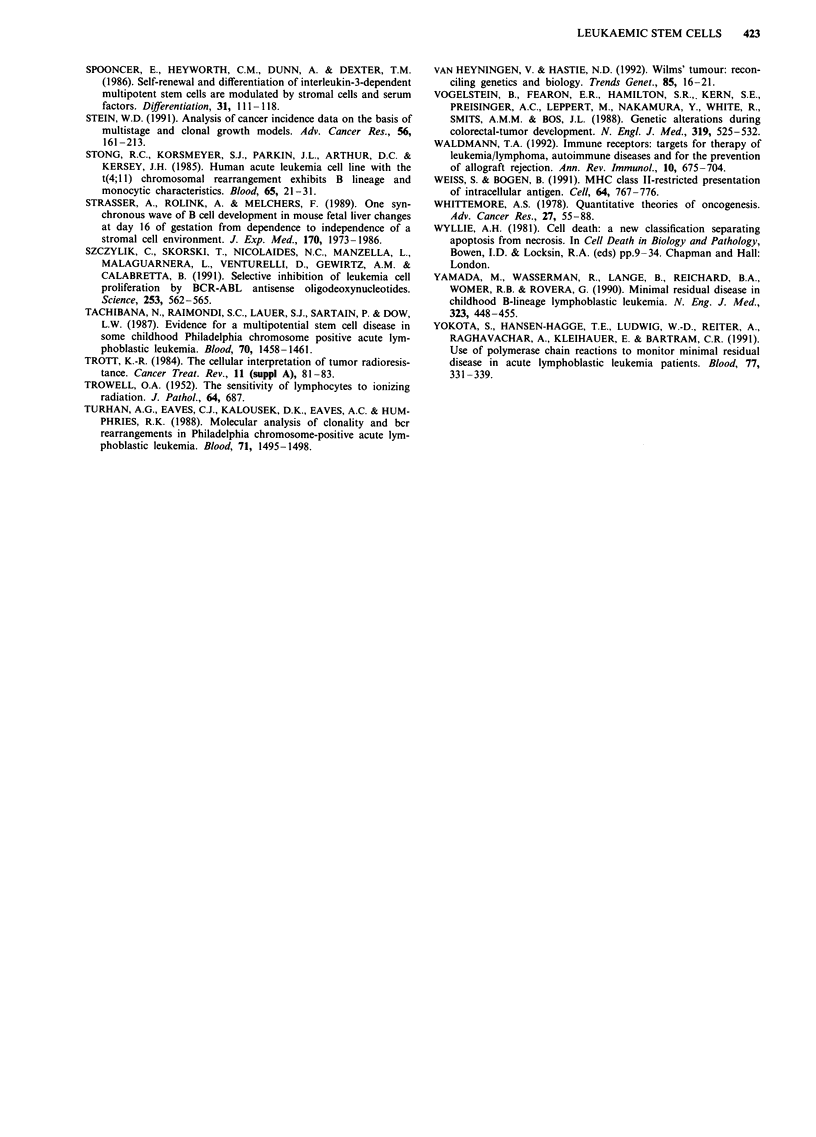# Stem cell origins of leukaemia and curability.

**DOI:** 10.1038/bjc.1993.81

**Published:** 1993-03

**Authors:** M. F. Greaves

**Affiliations:** Leukaemia Research Fund Centre, Institute of Cancer Research, Chester Beatty Laboratories, London, UK.

## Abstract

It is suggested that most childhood acute lymphoblastic leukaemias and some other paediatric cancers are chemo-curable because they arise in stem cell populations that are functionally transient, chemosensitive and programmed for apoptosis. Most adult acute leukaemias are chemo-incurable at least in part because they originate in relatively drug resistant stem cells with extensive self-renewal capacity. The latter property in turn increases the probability of clones evolving with multi-drug resistance. Particular mutations may superimpose additional adverse features on leukaemic cells.


Br. J. Cancer (1993), 67, 413–423 © Macmillan Press Ltd., 1993

HYPOTHESIS

### Stem cell origins of leukaemia and curability

M.F. Greaves

*Leukaemia Research Fund Centre, Institute of Cancer Research, Chester Beatty Laboratories, 237 Fulham Road, London SW3 6JB, UK.*

**Summary** It is suggested that most childhood acute lymphoblastic leukaemias and some other paediatric cancers are chemo-curable because they arise in stem cell populations that are functionally transient, chemo-sensitive and programmed for apoptosis. Most adult acute leukaemias are chemo-incurable at least in part because they originate in relatively drug resistant stem cells with extensive self-renewal capacity. The latter property in turn increases the probability of clones evolving with multi-drug resistance. Particular mutations may superimpose additional adverse features on leukaemic cells.

### Curability of childhood leukaemia

Childhood acute lymphoblastic leukaemia (ALL) has provided a landmark in cancer therapy as the first disseminated and otherwise lethal malignancy to be curable in the majority of patients. This success originated over a 30 year period, despite a tide of deep pessimism, with the introduction by Farber *et al.* (1956) of single agent drugs to induce temporary remissions followed by the elaboration of combination chemotherapy for both induction and maintenance of remission, the addition of prophylactic CNS radiation to cope with the major extramedullary sanctuary and the intensification of treatment for higher risk patients. This accumulative progress over 40 years now provides an expectation of very long term remission and probable cure in approximately 75% of children with ALL who have access to the best treatment (Pinkel, 1979; Simone, 1979*a*; Gale & Hoelzer, 1990; Riehm, 1991).

The hope that this pattern of progress and the strategies used could be built upon to improve treatment and outcome in other cancers has met with only partial and very selective success. Thus whilst several of the paediatric cancers including, in particular, Hodgkin's disease and Wilms' tumour now have very high cure rates of around 90% (Pochedly, 1987; Crist & Kun, 1991), amongst adult disseminated or metastatic cancers the story remains dismal, testicular teratoma providing one of the few examples of effective treatment (Einhorn, 1990). In marked contrast also to leukaemia in childhood, acute leukaemias in adults have proven much more intransigent to combination chemotherapy; the best estimates of cure rates for adult (> 15 years) ALL and acute myeloid leukaemia (AML) are around 30% (Gale & Hoelzer, 1990; Gale, 1990). Some optimism still remains that this picture will improve with newer or more intensive regimes of induction chemotherapy for adult leukaemias, but it is clear that a striking difference in clinical response to chemotherapy exist between these different cancers in association with age, cancer type or both.

Several factors could collectively account for this pattern of selective curability. First, it could be argued that the very systematic collaborative and multi-disciplinary approach to the development and application of multi-agent chemotherapeutic strategies and supportive care for childhood cancers has not really been taken on board for most adult cancers. This seems very unlikely to be the explanation. Second, children may be diagnosed and receive systemic therapy at a relatively early stage in disease progression. Third, children may have much better vital tissue regenerative capacity than adults enabling them to withstand and recover from toxic side-effects of intensive treatment. The latter is almost certainly a relevant factor with respect to the substantial fraction of patients over 60 years of age with leukaemia or cancer. This is unlikely to be the whole explanation, however, since some childhood cancers remain difficult to treat successfully (e.g. metastatic neuroblastoma; infant leukaemia) and some adults are successfully cured.

Physicians who have played prominent roles in the treatment of childhood leukaemia have suggested possible explanations for the effectiveness of chemotherapy for paediatric ALL. Simone has suggested that the disease (or leukaemic cell population involved) is in some way *intrinsically* sensitive to chemotherapy as evidenced by dramatic responses to different drugs given as single agents (Simone, 1979*b*). Operationally speaking, Simone must be correct but this does not provide us with a biological explanation. A somewhat different view is held by Pinkel (1987). He suggests that the genetic alterations underlying leukaemic cell transformation disrupt cell phenotype to an extent that a clear relationship to normal cells cannot be ascertained. Efficacy of treatment, he suggests, somehow relates to the ability of the drugs used to induce clinical remission by killing the bulk of tumour but cure of the disease is achieved by drugs used in maintenance of remission (e.g. methotrexate) that are capable of neutralizing or reversing the abnormal genetic programme in remaining leukaemic cells allowing them to respond to normal physiological signals promoting differentiation. It follows from this, Pinkel argues, that therapy should be dictated by, and targeted to, the genotype of the leukaemia (rather than presumed cell type) and cites as evidence the correlation between clinical outcome and presence of particular chromosome translocations and/or oncogene alterations.

It is self-evident that genetic alterations in cancer will indeed modify cell phenotype, differentiation competence, growth rates, probability of subsequent mutations, and concomitantly influence susceptibility of the leukaemic cell population to drugs. In this article, I am proposing that these effects, though important, are superimposed upon more fundamental mechanisms that provide the primary determinant of curability. The explanation suggested here, as a hypothesis, runs contrary to Pinkel's views only in so far as properties of the cell type in which cancer originates are considered to be of importance with respect to subsequent clinical response and that curable and incurable leukaemias may on the whole, though not exclusively, originate in different cell types. The theory is relevant also to a consideration of other 'curable' paediatric cancers. Adult cancers other than leukaemias are not considered except to point out that the unusual curability of one of them may owe its success to certain features shared with lymphoid progenitor cells (see Addendum).

Correspondence: M.F. Greaves, Leukaemia Research Fund Centre, Institute of Cancer Research, Chester Beatty Laboratories, 237 Fulham Road, London SW3 6JB, UK.
Received: 22 July 1992; and in revised form 12 October 1992.

**When is a stem cell a stem cell?**

Stem cells are usually considered as long-lived and, perhaps, immortal cells that normally reside out of cycle, in Go, but can be induced to proliferate and either self-renew (i.e. produce another stem cell) over many repeat cycles (>200) (Lajtha, 1979; Potten & Loeffler, 1990) or produce differentiating progeny of one or more lineages. The alternative pathways are balanced overall to maintain steady state conditions. The only unequivocal examples of normal stem cells defined by these criteria are in tissues that self-renew throughout life, i.e. stratified squamous epithelia, male germ cells in the testes and the haemopoietic system (Lajtha, 1979; Hall & Watt, 1989; Potten & Loeffler, 1990). Many haemopoietic lineage diagrams show multi-potential stem cells giving rise to transitory lineage committed progenitor cells which themselves proliferate to produce maturing descendent cells but which, unlike stem cells, do not self-renew. In other words, self-renewal and differentiation are considered to be strictly compartmentalised and mutually exclusive. As others have argued (Potten & Loeffler, 1990), this rigid model is almost certainly incorrect. A more plausible scheme allows for stem cells to be a heterogeneous population and for self-renewal and differentiation to be inversely related in a progressive hierarchical manner rather than all or none and for the balance between these two properties to be regulated or determined by micro-environmental signals. Circumstances when otherwise transitory progenitors might self-renew and therefore express stem cell-like characteristics would include particular culture conditions *in vitro* (Spooncer *et al.*, 1986), during phases of regeneration *in vivo*, e.g. following recovery from toxic chemotherapy, radiation and/or transplantation (Potten & Loeffler, 1990) and during ontogeny (see below). Under these circumstances, however, self-renewal will be time limited. The existence of these temporary periods of stem cell-like behaviour are significant because they could denote periods during which these proliferating cell populations are at extra risk of mutation.

The sites of B and T lymphopoiesis (foetal liver/bone marrow and thymus respectively) are invaded, during ontogeny, by a few, time-limited waves of proliferating, stroma-dependent progenitor cells (Le Douarin, 1978; Strasser *et al.*, 1989). This developmental 'window' provides the opportunity required for clonal diversification of Ig/T cell receptor genes (Alt *et al.*, 1986) before further differentiation and clonal selection occurs. Although B cell production continues throughout life, proliferation in the precursor compartment is much more extensive in very young animals (Miller & Osmond, 1975). In the T cell system, the thymus atrophies in the post-pubertal period (Clarke & MacLennan, 1986) and fewer T cells are presumably processed thereafter.

Note that mature lymphoid cells are unique since as differentiated cells, they can express some stem cell properties including, in the case of T cells, life-spans (in interphase/Go) of decades, extensive self-renewal and proliferative capacities. It is probably for this reason that lymphocytes alone amongst mature or fully differentiated cells are vulnerable to malignant transformation.

Lymphopoiesis is also characterised by very extensive apoptosis, an active process involving protein synthesis, endonuclease activation and DNA fragmentation (Wyllie, 1981). Moreover, most lymphocytes are very sensitive to DNA-damaging agents, a feature they share with few other cell types tested, with the interesting and relevant exception of male germ cells. For example, mature lymphocytes are very unusual in being susceptible to interphase death with ionising ($\gamma$, X)-radiation (Maruyama & Feola, 1987) and are very sensitive to steroid-induced apoptosis (Wyllie, 1981). Lymphocyte progenitor populations may be ultra-sensitive to DNA damage. The extreme sensitivity of cortical thymocytes (T cell precursors) to steroids and $\gamma$-radiation has been recognised for decades (Trowell, 1952), the lethal effects in both cases appearing to follow the apoptotic pathway (Sellins & Cohen, 1987). Recent observations indicate that clonogenic B cell progenitors have a unique sensitivity to ionising radiation equalling or surpassing that of cells that are mutants for DNA repair (Griffiths *et al.*, in preparation). These cells are killed by a single passage of $\alpha$-particles (plutonium-238) and have a Do for x-rays of $\sim$0.3 Gy. The basis of this intrinsic vulnerability of lymphoid cells may include the accessibility of the apoptosis programme (Wyllie, 1981; Griffiths *et al.*, in preparation) and a reduced ability to repair double stranded DNA breaks (Mayer *et al.*, 1986). In these respects lymphoid progenitors are considerably more susceptible than myeloid progenitors or multi-potential haemopietic stem cells (Goldschneider *et al.*, 1979; Griffiths *et al.*, in preparation); indeed, a substantial fraction of the latter are remarkably resistant to a wide range of drugs. Several mechanisms could contribute to stem cell resistance, including effective DNA repair capacity, residence out of cycle with concomitant loss of dependence on growth factors or vulnerability to apoptosis (Leary *et al.*, 1989), and high level expression of gene products associated with drug resistance including the multi-drug efflux pump P glycoprotein (Chaudhary & Roninson, 1991) and aldehyde dehydrogenase (Kastan *et al.*, 1990).

Stem cells have been regarded as the principal or exclusive 'target' cell population for the initiation of malignancy since their longevity provides the requisite opportunity for subsequent independent mutations within the same clone whilst accommodating the principles of latency and differentiation arrest (Cairns, 1975; Buick, 1987; Pierce & Speers, 1988; Potten & Loeffler, 1990). Transit cells, by the same criteria, have been considered poor or ineffective targets. Clearly, these boundaries of vulnerability change when a less structured definition of stem-ness is allowed, especially for tissues such as the lymphoid system.

With respect to malignancy in the haemophoietic system, therefore, cells at three development stages (Figure 1) are potentially at risk of mutation and clonal selection leading to leukaemia or other blood cell malignancies (lymphoma, myeloma). Rarely, cells ancestral to the haemopoietic system including embryonic germ cells (Nichols *et al.*, 1990) or the parental germline (Felix *et al.*, 1992) may also provide targets for initiating mutations that lead to leukaemia.

**An hypothesis**

Extensive studies on radiation treatment and chemotherapy, both in animal model systems and in human cancer have suggested that one key factor in determining curability or resistance is likely to be the total burden of clonogenic or stem cells in the tumour, these cells being responsible for sustaining tumour growth and hence the relevant target population for therapy (Skipper & Schabel, 1984; Trott, 1984; McGuire *et al.*, 1985, Buick, 1987). In acute leukaemia, this association is reflected in the correlation between *in vitro* clonogenicity and adverse prognosis (McCulloch *et al.*, 1982). It follows from this that the intrinsic stem cell properties of the 'target' cells for different types of leukaemia or cancer might be expected to have an impact on curability. Based on these considerations, an hypothesis to explain the marked difference in curability between acute leukaemia in children and adults is as follows.

(1) The major 'target' cells for leukaemia in children are B lymphoid (and to a lesser extent T and myeloid) *committed* progenitor cells (Greaves, 1986). During a limited period of early development, these cells have extensive self-renewal capacity regulated by specialised stromal/growth factor conditions in foetal liver and, subsequently, bone marrow (Kincade, 1987; Rolink & Melchers, 1991) but they differ from true stem cells by their finite self-renewal capacity and their low probability of an exit from cycling, i.e. they are still transitory cells. These same cells are programmed for cell death (apoptosis) and can only be rescued by finding the correct developmental niche (stroma/growth factors) that facilitates proliferation and/or differentiation (plus they must achieve functional Ig/TCR gene rearrangement) (Hardy *et al.*, 1991*a*). Leukaemic transfor-

**Figure 1** Development associations of leukaemia and stem cells.
†, cell death. S, DNA synthesis, cell proliferation. Go, non-cycling cells. L = lymphoid. M = myeloid. + + + = extensive self-renewal capacity; + + + → − (age) = variable self-renewal capacity (associated with age) from extensive (+ + +) to zero (−).

mation of these cells may be facilitated by the intrinsic mutagenicity of the recombinases (Fuscoe *et al.*, 1991) and TdT (Kunkel *et al.*, 1986) that are involved in immunoglobulin gene diversification (Lieber, 1992; Schatz *et al.*, 1992). The leukaemic (B precursor) progeny of the clonogenic cells in acute lymphoblastic leukaemia (ALL) inherit ultra-sensitivity to apoptosis-inducing drugs or ionising radiation. The crucial corollary is that in most cases, the leukaemogenic mutations are not in genes that, when dysregulated, can block apoptosis. The clonogenic cells, though potentially immortal, are a very minor fraction of the total leukaemic cell population but, initially at least, they too are also very sensitive to the lethal effects of drugs and ionising radiation. Killing of the great majority, or all, of these leukaemic cells and potential cure is therefore possible *provided* therapy is adequately delivered prior to the emergence of subclones that are mutants for drug uptake or apoptosis. A minority of patients presenting with a common ALL phenotype but with a very high white cell count at diagnosis are likely to be at a significant disadvantage in this respect. Normal B cell progenitors will also be obliterated but the potentially hazardous sequelae of such a loss will be avoided as this cell population is replenished from the largely intact and relatively drug resistant pool of lympho-myeloid stem cells.*

(2) The majority of adult acute leukaemias (ALL, AML and acute phase CML) and a minority of childhood leukaemias, originate in more primitive multi-potential stem cells. These are major targets in adults because the extended time scale involved (decades) provides the opportunity for accumulation of two or more mutations in cells that divide infrequently. The clonogenic cell population in these leukaemias, in contrast to childhood ALL, are resistant to complete ablation by chemotherapy because (i) their inherently greater self-renewing capacity, constitutively expressed following transformation, results in a larger total burden of clonogenic cells. They can only be completely eliminated by killing or damaging normal haemopoietic cell stem cell populations below the level of tolerance; (ii) they may be inherently less sensitive as individual cells to DNA damaging agents then clonogenic lymphoid progenitors (see above). *Progeny* of these clonogenic leukaemic stem cells may nevertheless express the more susceptible phenotype of differentiated progenitor/precursor populations; clinical, but not biological, remission can therefore be achieved, and. (iii) the larger total burden of accumulated stem cells substantially increases the likelihood that drug resistant mutants will exist by the time that treatment is initiated (see Table I).

Two important caveats are as follows:

(1) Butturini and Gale argue (1989*a*) that the age dependent pattern of leukaemic subtypes is a feature of exposure to particular aetiological agents or pathways rather than a reflection of intrinsic changes in cell populations with age. The above hypothesis is not defined in terms of aetiological agents but is certainly compatible with an interactive model in which both cell population characteristics and exposure to hypothetical aetiological factors may be rate limiting and time constrained. For example, the common (and curable) variant of childhood ALL has been suggested

*This interpretation supposes that cure is due to elimination of effectively all clonogenic ALL cells. Recent studies in which residual disease in long term remission is assayed by PCR for clone specific IgH rearrangements are compatible with this view (Yamada *et al.*, 1990; Yokota *et al.*, 1991). It is, however, striking in these studies that leukaemic cells persisted for up to 18 months in patients who remain in long term remission.

**Table 1** Characteristics of haemopoietic cells that may influence leukaemogenesis and treatment outcome

| | | *Features that influence vulnerability to leukaemogenesis ( + or – )* | *Features that influence chemo-curability of leukaemias ( + or – )* | *Malignancies* |
|---|---|---|---|---|
| 1 | Multi-potential stem cells | Majority out of cycle ( – )<br>Smaller number ( – )<br>Extensive self-renewal capacity (and longevity as clone)<br>( + over long time-frame) | Intrinsically chemo-resistant, plus acquired resistance through extensive self-renewal ( – )<br>Proportionally high content of clonogenic cells ( – )<br>Irreplaceable ( – ) | CML<br>Most adult AML, ALL |
| 2 | Lineage-restricted progenitor cells | Majority in cycle,<br>large numbers during developmentally timed periods,<br>finite self-renewal (developmentally timed) ( + , in early development, decreasing risk with time) | Chemo-sensitive ( + )<br>Proportionally low content of clonogenic cells ( + )<br>Replaceable ( + ) | Most childhood acute leukaemias |
| 3 | Mature cells: | | | |
| | Myeloid | Dying end cells ( – ) | | None |
| | Lymphoid | Majority out of cycle ( – ) but periodically activated by antigen ( + )<br>Large numbers ( + )<br>Extensive self-renewal capacity ( + ) | Chemo-sensitive ( + ) but acquired resistance through extensive self-renewal ( – )[a]<br>Replaceable ( + ) | Chronic lymphoid leukaemias<br>Non-Hodgkin's lymphoma<br>Myeloma |

[a]Or resistance induced by *BCL*-2 rearrangements (see Appendix).

to arise in part as a result of altered patterns of infection in infancy (Greaves, 1988). Recent epidemiological evidence provides some persuasive, if indirect, support for this view (Alexander *et al.*, 1990; Kinlen *et al.*, 1990; Draper *et al.*, 1991; reviewed in Greaves & Alexander, 1992).

(2) Even within what appears to be an intrinsically curable cancer such as childhood ALL, prognostic variables have been identified in the past including total white cell count and other indicators of 'tumour' load but also cell type and karotypic markers. High white cell count is a highly significant and independent indicator of poor prognosis. A minority of patients with B cell precursor (common) ALL present with high counts, as do a more substantial proportion of the less common T-ALL. These patients usually obtain remission but frequently relapse, indicating perhaps that a high tumour burden is likely to be associated with a larger pool size of clonogenic cells and hence a higher probability of drug resistant mutants. Common ALL with hyperdiploidy usually presents with modest white cell counts and require less intensive treatment in order to induce and sustain long term remissions than cytoplasmic $\mu$ chain positive B precursor subset of common ALL or those with T cell phenotypes (Pui *et al.*, 1990). With more effective treatment schedules, some of these prognostic correlates may lose significance (Pui *et al.*, 1990). This variability could be dependent upon intrinsic features of the particular cell type involved or variation in the timing of diagnosis in relation to the natural history of the disease. It may also, in some cases at least, reflect the super-imposition of adverse leukaemic features per se as argued by Pinkel. The Philadelphia chromosome positive leukaemias are especially interesting in this regard.

**Clonogenic origin, cellular phenotype and curability: lessons from Ph-positive leukaemias**

The hypothesis proposed here had its historical origins in a consideration of the remarkable difference in curability of two leukaemias that appeared to involve the same cell type (Greaves, 1982); these are the common (c) variant of acute lymphoblastic leukaemia and lymphoid blast crisis of CML. The leukaemic cells in each leukaemia have a B lineage precursor phenotype with clonal rearrangements of IgH genes (Greaves *et al.*, 1979; Bakhshi *et al.*, 1983; Ford *et al.*, 1983; Greaves, 1986). Common ALL represents the high cure rate subgroup of ALL in children (Chessells *et al.*, 1977; Sallan *et al.*, 1980; Greaves *et al.*, 1981). On the basis of the equivalent morphology and immunophenotype, patients with lymphoid blast crisis of CML were treated with chemotherapeutic regimes appropriate for ALL (Marks *et al.*, 1978; Janossy *et al.*, 1979). Remission was achieved in a majority of patients; these were, however, relatively short-lived and few, if any, patients with lymphoid or myeloid blast crisis of CML survive for more than 2 years (Shaw, 1982). Since it was already known by that time (by lineage analysis of clonal markers) that CML was a lympho-myeloid stem cell disease (Fialkow, 1980), the explanation offered for the marked difference in curability was that illustrated in Figure 2. This model predicts that comparable lymphoid precursor phenotypes of the 'bulk' leukaemia cell population will be paralled by remission induction but that the critical difference in cure in the two leukaemias resides in distinct clonogenic stem cell origins.

The evidence that most cALL in children does, as predicted, originate in B lymphoid progenitor cells rather than multi-potential stem cells, rests on the observation that myeloid cells in such patients are not demonstrably part of the leukaemic clone as indicated by the use of X-linked polymorphisms as markers – glucose 6-phosphodehydrogenase (Dow *et al.*, 1985), HPRT and PGK (Greaves *et al.*, 1991; Ford, Pegram and Greaves; unpublished data). More conclusive evidence could be obtained now from analysis of individual myeloid cells or colonies by combined genotype (FISH)/immunophenotype methods (Price *et al.*, 1992) or by PCR (Hernandez *et al.*, 1990). It will be important to apply these methods also to the subset of adult ALL that are curable and therefore predicted to be lymphoid restricted in origin.

These data are in accord with the restricted stem cell hypothesis for curable childhood leukaemia but suffer from the fact that they do not accommodate the 'Pinkel' interpretation, i.e. since CML in lymphoid blast crisis have a *BCR/ABL* (p210) rearrangement (plus other genetic alterations), which most childhood ALL do not have, how can we rule out that it is this acquired molecular feature which determines the incurability of CML in lymphoid blast crisis? This point becomes even more significant and interesting when ALL presenting with the Ph chromosome (i.e. no preceding chronic phase or CML) are considered (Beard *et al.*, 1976; Peterson *et al.*, 1976). These cases usually have a B cell precursor immunophenotype (Janossy *et al.*, 1978) equivalent to that of Ph-negative cALL, although some have mixed lineage or 'lympho-myeloid' characteristics (Hirsch-Ginsberg *et al.*, 1988). Ph-positive ALL have an extremely poor prognosis (Bloomfield *et al.*, 1986; Ribeiro *et al.*, 1987; Pui *et al.*, 1990); few, if any, are curable by chemotherapy. In

**Figure 2** Relationship between clonogenic 'target' cells and chemotherapeutic cure in ALL versus ALL-like lymphoid blast crisis of CML. c = cell death induced by chemotherapy. d = differentiation arrest. 1°, 2°, sequential genetic events (in CML). X, genetic event(s) giving rise to common ALL ($Ph^-$). Lymphoid blast crisis of CML could, in theory, arise by mutations in either a (Ph-positive) committed lymphoid stem cell/progenitor cell, or (as shown in Figure 2) a (Ph-positive) lympho-myeloid stem cell whose progeny then preferentially and partially differentiate into early (B) lymphoid cells. Either way, transient haematological remission might be expected because of the intrinsic sensitivity of these progeny cells but this will not be sustained because of the existence of residual clonogenic cells in the more primitive lympho-myeloid stem cell compartment that are capable of resurrecting the blast crisis status.

a study from St Jude Hospital, there were only two out of 18 long term survivors of children with Ph-positive ALL; those two had variant translocations involving 22q11 (Ribeiro *et al.*, 1987). Subsequent studies have revealed that these variant forms may not involve detectable *BCR/ABL* kinase (Dow *et al.*, 1989) and are, therefore, distinct from classical Ph-positive leukaemias. The presence of the Ph chromosome appears to be prognostically significant, independent of other clinical parameters including white cell count (Secker-Walker *et al.*, 1991). Significantly, far more adult (~25%) than children (~3.5%) with ALL are Ph-positive (Bloomfield *et al.*, 1986; Ribeiro *et al.*, 1987; Pui *et al.*, 1990). The children with Ph-positive ALL are older than average (Ribeiro *et al.*, 1987). Recent studies indicate that the proportion of adult ALL that are Ph-positive also increases with age (Maurer *et al.*, 1991; Secker-Walker *et al.*, 1991). Age is itself a significant prognostic variable in ALL (Henderson *et al.*, 1990). Collectively, therefore, these data suggest that a major factor contributing to the substantial age-linked variation in chemo-curability of ALL is the incidence of Ph-positive disease (Figure 3).

If we accept that the common form of (Ph-negative) ALL in children might indeed be curable for the reasons suggested above, then the issue for chemo-incurability of Ph-positive ALL (and perhaps adult acute leukaemia in general) is whether this reflects a multi-potential stem cell origin, and therefore 'intrinsic' resistance or whether the *BCR/ABL* kinase (and other chromosomal/molecular abnormalities commonly found in Ph-positive ALL; Rieder *et al.*, 1991; Russo *et al.*, 1991) enables the leukaemic cell to over-ride intrinsic susceptibility (the Pinkel argument). The cellular origins of Ph-positive ALL are clearly germane to this issue. Could this be a lympho-myeloid stem cell leukaemia despite its predominant B cell precursor phenotype? When Ph-positive ALL was first described (Beard *et al.*, 1976), it was suggested that these were in fact lymphoid blast crises evolving from clinically covert CML. That some patients reverted, following treatment, to a CML-like picture (Catovsky, 1979) lent strong support for this view. Attempts to determine the origin of Ph-positive ALL more directly by clonal analysis has, however, produced somewhat conflicting results.

Analysis of myeloid cell populations or colony-forming cells in some cases of Ph-positive ALL has indicated that they lack detectable Ph chromosome or *BCR/ABL* rearrangements (Kitano *et al.*, 1988; Craig *et al.*, 1990). These observations are open to several interpretations but taken at face value do not support a lympho-myeloid stem cell origin for Ph-positive ALL. In other cases of Ph-positive ALL, however, the Ph chromosome or its molecular lesion have been detected in myeloid cells providing formal proof of a multi-potential stem cell origin (Tachibana *et al.*, 1987; Turhan *et al.*, 1988; reviewed in Secker-Walker & Craig, 1993).

At present, therefore, it seems likely that the relatively high frequency of Ph-positive ALL in adults contributes very significantly to the chemo-incurability of most adult patients but that the two alternative explanations discussed above do not enjoy exclusive support. Possibly therefore a multi-potential stem cell origin and the presence of the *BCR/ABL* kinase can both contribute significantly to the intransigence of Ph-positive adult ALL to chemotherapy.

**Figure 3** Frequency of Ph-positive ALL with age. Data derived from references 75 and 78.

Similar arguments apply to childhood versus adult AML although here the Ph chromosome plays only a very minor role. In AML, there is evidence, again from analysis of clonality by X-linked polymorphisms, that the largely intractable disease in older adults may predominantly originate in a primitive multi-potential stem cell (Fialkow *et al.*, 1987). Additionally, several forms of clonal haemopoietic dysplasias in adults with a high probability of progressing to AML involve a common lympho-myeloid stem cell (Fialkow, 1984; Buschle *et al.*, 1988). AML in children requires more intensive chemotherapy than ALL and has an appreciably lower complete remission rate, remission duration and cure rate than childhood ALL (Kalwinsky *et al.*, 1988; Gale, 1990). However, long term remissions and possible cures are now obtainable in a substantial fraction (30–50%, cf 15–20% in adults) (Clarkson *et al.*, 1990; Rohatiner & Lister, 1990; Schellong *et al.*, 1990). In children and younger adults, the disease appears to be more frequently (though not exclusively) clonally restricted to a single (granulocytic) lineage indicating a possible origin from a more differentiated myeloid progenitor cell (Fialkow *et al.*, 1987) which may nevertheless be intrinsically less drug sensitive than their lymphoid counterparts. Interestingly, as in adult ALL, inferior prognosis in adult AML is very significantly correlated with increasing age (Gehan *et al.*, 1976; Rohatiner *et al.*, 1990). This still holds true if very old patients are excluded. These observations are compatible with the view that paediatric AML arising in myeloid committed progenitor cells are more drug sensitive than those arising in more primitive lympho-myeloid stem cells, but are nevertheless less sensitive to eradication than paediatric ALL cells because they do not share the critical attributes identified in lymphoid progenitor cells. Direct evidence for this view comes from recent studies comparing the sensitivity of clonogenic B cell precursor versus myeloid cells to ionising radiation and apoptosis-inducing drugs dexamethasone, cisplatinum and etoposide (Griffiths *et al.*, in preparation).

Collectively then, these observations are largely compatible with the model proposed. However, at present, the evidence is incomplete and alternative explanations cannot be entirely ruled out.

### Age related cancer incidence rates and stem cell origins

Figure 4 illustrates a comparison of age related incidence rates of three different malignancies. Aside from the enormous numerical differences in incidence rates, the striking feature is the shape of the incidence rate curves which are

**Figure 4** Age related cancer incidence rates.
ALL, osteosarcoma = annual incidence rate per $10^5$.
Lung cancer = annual incidence rate per $10^3$.

accumulative or exponential for epithelial carcinoma but more age restricted, normal or bimodal in distribution for cALL and osteosarcoma (as for most paediatric cancers) (Pochedly, 1987). Note also that the incidence rates of the two examples of childhood tumours chosen have very different age distributions. As predicted by epidemiological (Armitage & Doll, 1954) and mathematical considerations (Whittemore, 1978; Stein, 1991) and endorsed by recent molecular biological evidence (Vogelstein *et al.*, 1988), the adult curve probably reflects the required accumulation of rare successive mutations in long lived epithelial cells, the risk of a 'full house' thereby increasing with time. In children, the distribution suggests a developmentally regulated and restricted risk period which is different for different cell types. The incidence rate profile of osteosarcoma is paralleled in adolescent boys and girls by the post-pubertal spurt of long bone growth (also the common site of origin of this tumour) (Meyers, 1987). The simplest explanation of the age distributions in paediatric cancers is therefore that these reflect developmentally restricted windows of proliferative stress for particular tissue specific progenitor cell types. Given that at least two rate limiting mutations are probably required

(Knudson, 1977; Graf, 1988; Haber & Housman, 1991), the incidence rate at any age in children is likely to reflect the probability of the final or rate limiting mutation occurring. In the simplest two-step model, therefore, the earlier or first mutation could occur at any proceeding time in the same or an antecedent cell type. In the case of the common (c) variant of B cell precursor childhood ALL, it was suggested earlier that the very marked age associated peak of maximum incidence (2–5 years) (Greaves *et al.*, 1985) reflected the timing of a penultimate mutation promoted by proliferative stress associated with infection (Greaves, 1988). This event was further predicted to occur in a pre-leukaemic clone initiated *in utero*.

The concept that paediatric cancers are genetic abnormalities of development is not new but there have been few attempts to rationalise this idea and contrast it with adult cancers in the context of the biology of tissue specific stem cells and lineage pathways.

One obvious interpretation of the highly restricted distribution of the paediatric tumours is that beyond the time-frame of observed disease, the relevant cell types are effectively not at risk either because their numbers (as proliferating cells) are very small or nonexistent and/or specialised micro-environmental conditions for their growth no longer exist. This would clearly be compatible with the morphogenic programme of non-self-renewing tissue such as muscle or nerve, compared with most epithelial tissues which must continually replenish. Embryonic cells which form these structures exist as a developmental wave of progenitors, e.g. metanephric blastemal cells in the foetal kidney (Van Heyningen & Hastie, 1992). They need no constitutive stem cell properties but rather self-renewing capacity which is *temporarily* expressed then either exhausted or severely restrained by the developing tissue itself. The progenitor cells in developmental pathways in non-renewable tissues may therefore be programmed for finite or restrained self-renewing properties. Additionally, as in the lymphoid system, they are associated with a remarkable degree of apoptosis or programmed cell death (Hinchliffe, 1981). It follows from this that unless the genetic mechanisms underlying neoplasia radically change these properties, then a corresponding population of malignant clonogenic cells, as well as their descendant progeny (i.e. in paediatric solid tumours), would, initially at least, be very susceptible to varying combinations of drugs and/or radiation for the same reasons as the lymphoid progenitor cells that give rise to childhood ALL.

### Implications for therapy

One clinical implication of the hypothesis discussed here is that a substantial fraction of adult leukaemias may be intrinsically chemo-incurable. Sceptics of this view will point to the historical perspective on childhood leukaemia and recent, albeit modest, success in treating adult leukaemia and conclude that more intensification of treatment, better drugs (cf platinum derivatives in ovarian carcinoma (Hardy *et al.*, 1991*b*) or judicious combination of drugs will eventually do the trick. This optimistic view still prevails but there appears to be inadequate evidence to sustain it. The alternative position is to accept the intractable nature of adult acute leukaemia and the inherent difficulty of identifying 'conventional' drugs that will discriminate effectively between normal and leukaemic stem cells and to vigorously pursue different therapeutic strategies. Those currently used or under development include allogeneic bone marrow (stem cell) transplantation as a rescue device for supra-lethal treatment of patients (Champlin & Gale, 1991). This is in theory the logical manoeuvre for a multipotential stem cell disease but has obvious limitations of donor compatibility and inherent risks of failure or serious side effects. The practicality and efficacy of this approach may in the future be enhanced by deriving matched donor stem cells from extensive cord blood banks (Broxmeyer *et al.*, 1989) and the use of recombinant growth factors to facilitate speedy haemopoietic reconstitution (Metcalf, 1985). Other 'biological' approaches to potential cure include the use of growth factors to 'differentiate-out' the leukaemic clone (Metcalf, 1985; Fenaux *et al.*, 1992), anti-sense oligonucleotides (McManaway *et al.*, 1990; Reed *et al.*, 1990; Calabretta, 1992) that could be targeted to leukaemia-specific DNA or mRNA sequences, such as the BCR/ABL junction (Szczylik *et al.*, 1991), and immunotherapy. The latter approach has a somewhat inglorious history in leukaemia and cancer treatment but is undergoing a renaissance within a more logical framework with the demonstration of a graft versus leukaemia effect in transplanted patients (Butturini & Gale, 1989*b*) and, in particular, the appreciation that products of deregulated genes, e.g. IL2 receptor (Waldmann, 1992) and mutated or fused oncogenes encoding novel gene products, might serve as targets for antibody or T cell mediated immunological attack. The latter approach might seem implausible for intracellular proteins such as BCR/ABL or mutated RAS but may in fact be possible if, as demonstrated in other systems, intracellular proteins are catabolised and processed to produce peptides that cycle within the cell and associate with cell surface MHC molecules (Weiss & Bogen, 1991; Neefjes & Ploegh, 1992). This view is much encouraged by the demonstration that T cells from mice injected with synthetic peptides can specifically recognise mutated RAS protein (Jung & Schluesener, 1992) and the BCR/ABL fusion protein (Chen *et al.*, 1992).

### Addendum: exceptions that may prove the rule

### *Infant acute leukaemias*

Infant ($<18$ months) acute leukaemias occur at a very low incidence rate but have a particularly poor prognosis (Crist *et al.*, 1986; Pui *et al.*, 1990). This may in part be explained by the difficulties of treating such young individuals but features of the leukaemic cell populations are also likely to be relevant. The subtypes of acute leukaemia at this age are different from those of older children with a higher fraction having myeloid (including megakaryoblastic) features (Gardembas-Pain *et al.*, 1991). Some of those classified as ALL may be cryptic erythroleukaemias (Greaves *et al.*, 1983) and some may have monocytoid plus lymphoid characteristics (Stong *et al.*, 1985). The majority of infant ALL, however, have a B cell precursor phenotype (but lacking the CD10 marker of B precursor disease in older children) with clonal or oligoclonal rearrangements of the IgH gene and a high frequency ($\sim 75\%$) of chromosome translocations involving breaks at 11q23 (Raimondi *et al.*, 1989; Gibbons *et al.*, 1990; Pui *et al.*, 1990). Infant ALL usually presents with high white cell counts and CNS involvement, both indicative of a high 'tumour' burden. There is currently no data available on the clonal origins of these leukaemias. The prediction which requires testing is that by birth in such cases, at least one of the necessary two or more genetic events (including $11q23^-$) have already occurred and that the disease originates in primitive stem cells that are distinct from those involved in common ALL.

### *Non-Hodgkin's follicular lymphoma*

This tumour of mature B cells is the commonest lymphoid malignancy in Western countries. It is something of an enigma since it commonly presents (and persists for many years) as a low grade benign disease which can be controlled but not erradicated by treatment and is almost invariably fatal following eventual progression to high grade diffuse disease after 10 years or so (Magrath, 1990). The majority of follicular (centroblastic/centrocytic) lymphomas ($>75\%$) have a chromosomal translocation which results in dysregulation of the gene *BCL*-2 by association with the IgH locus (Bakhshi *et al.*, 1985). In this respect its natural history is not dissimilar to CML. The *BCL*-2 gene encodes a 24 kd mitochondrial membrane protein with the intriguing property of inhibiting the apoptotic programme (Hockenbery *et al.*, 1990) that is a normal feature of immunological regulation in germinal centre B cells (Liu *et al.*, 1991). Constitutive expression of *BCL-2* in transgenic mice endows mature B cells with a long or indefinite life-span, which is interpreted as protection from apoptosis (McDonnell *et al.*, 1989). It has been proposed that many chemotherapeutic agents used in cancer operate by inducing apoptosis (Eastman, 1990). Follicular lymphoma may then provide one example that fits Pinkel's assertion that genetic abnormalities determine treatment response since high level expression of bcl-2 protein might be expected to be protective. It will be interesting to see if other mutated or dysregulated genes in leukaemia, including *BCR/ABL* (Daley & Baltimore, 1988) and p53 (Lane, 1992), provide an escape from apoptosis independently of, or concomitant with, their proliferation effects.

### *Testicular teratomas*

These tumours occur in young adults and are remarkably radio- and chemo-sensitive resulting in high cure rates overall and including Stage IV patients with extensive metastases (Peckham, 1981; Einhorn, 1990; Roth & Nichols, 1992). Several different single agent drugs (e.g. actinomycin D) are effective in inducing remission although combined drug regimes are preferred (for advanced stages) recalling the earlier experience with childhood ALL. The cell of origin has not been identified but is presumed to be an early cell in the spermatogenesis pathway (Peckham, 1981). It would be a remarkable coincidence if the efficacy of both radiation and chemotherapy in this cancer were not associated with the high sensitivity of normal male germ cells to DNA-damaging agents (Meistrich & Van Beel, 1990), an unusual feature they share with lymphocyte progenitor cells.

I am grateful to Professor T.A. Lister, Dr G.J. Morgan and Dr W.M. Crist for helpful comments, the Leukaemia Research Fund of Great Britain for support and Ms Barbara Deverson with help in preparation of the manuscript.

### References

ALEXANDER, F.E., RICKETTS, T.J., MCKINNEY, P.A. & CARTWRIGHT, R.A. (1990). Community lifestyle characteristics and risk of acute lymphoblastic leukaemia in children. *Lancet*, **336**. 1461-1465.

ALT, F.W., BLACKWELL, T.K., DEPINHO, R.A., RETH, M.G. & YANCOPOULOS, G.D. (1986). Regulation of genome rearrangement events during lymphocyte differentiation. *Immunol. Rev.*, **89**, 5-30.

ARMITAGE, P. & DOLL, R. (1954). The age distribution of cancer and a multi-stage theory of carcinogenesis. *Brit. J. Cancer*, **8**, 1-12.

BAKHSHI, A., JENSEN, J.P., GOLDMAN, P., WRIGHT, J.J., MCBRIDE, O.W., EPSTEIN, A.L. & KORSMEYER, S.J. (1985). Cloning the chromosomal breakpoint of t(14;18) human lymphomas; clustering around $J_H$ on chromosome 14 and near a transcriptional unit on 18. *Cell*, **41**, 899-906.

BAKHSHI, A., MINOWADA, J., ARNOLD, A., COSSMAN, J., JENSEN, J.P., WHANG-PENG, J., WALDMANN, T.A. & KORSMEYER, S. (1983). Lymphoid blast crises of chronic myelogenous leukemia represent stages in the development of B-cell precursors. *N. Engl. J. Med.*, **309**, 826-831.

BEARD, M.E.J., DURRANT, J., CATOVSKY, D., WILTSHAW, E., AMESS, J.L., BREARLEY, R.L., KIRK, B., WRIGLEY, P.F.M., JANOSSY, G., GREAVES, M.F. & GALTON, D.A.G. (1976). Blast crisis of chronic myeloid leukaemia (CML). I. Presentation simulating acute lymphoid leukaemia (ALL). *Brit. J. Haematol.*, **34**, 167-178.

BLOOMFIELD, C.D., GOLDMAN, A.I., ALIMENA, G., BERGER, R., BORGSTRÖM, G.H., BRANDT, L., CATOVSKY, D., DE LA CHAPELLE, A., DEWALD, G.W., GARSON, O.M., GARWICZ, S., GOLOMB, H.M., HOSSFELD, D.K., LAWLER, S.D., MITELMAN, F., NILSSON, P., PIERRE, R.V., PHILIP, P., PRIGOGINA, E., ROWLEY, J.D., SAKURAI, M., SANDBERG, A.A., SECKER WALKER, L.M., TRICOT, G., VAN DEN BERGHE, H., VAN ORSHOVEN, A., VUOPIO, P. & WHANG-PENG, J. (1986). Chromosomal abnormalities identify high-risk patients with acute lymphoblastic leukemia. *Blood*, **67**, 415-420.

BROXMEYER, H.E., DOUGLAS, G.W., HANGOC, G., COOPER, S., BARD, J., ENGLISH, D., ARNY, M., THOMAS, L. & BOYSE, E.A. (1989). Human umbilical cord blood as a potential source of transplantable hematopoietic stem/progenitor cells. *Proc. Natl. Acad. Sci. USA*, **86**, 3828-3832.

BUICK, R.N. (1987). Biological and clinical implications of the stem cell concept in human malignancy. In *Cancer Biology and Therapeutics*, Cory, J.G. & Szentivanyi, A. (eds) pp.65-77. Plenum Press: New York.

BUSCHLE, M., JANSSEN, J.W.G., DREXLER, H., LYONS, J., ANGER, B. & BARTRAM, C.R. (1988). Evidence for pluripotent stem cell origin of idiopathic myelofibrosis: clonal analysis of a case characterized by a N-ras gene mutation. *Leukaemia*, **2**, 658-660.

BUTTURINI, A. & GALE, R.P. (1989*a*). Age of onset and type of leukaemia. *Lancer*, **ii**, 789-791.

BUTTURINI, A. & GALE, R.P. (1989*B*). Annotation: How can we cure leukaemia? *Brit. J. Haematol.*, **72**, 479-485.

CAIRNS, J. (1985). Mutation selection and the natural history of cancer. Nature, **255**, 197-200.

CALABRETTA, B. (1991). Inhibition of protooncogene expression by antisense oligodeoxynucleotides: biological and therapeutic implications. *Cancer Res.*, **51**, 4505-4510.

CATOVSKY, D. (1979). $Ph^1$-positive acute leukaemia and chronic granulocytic leukaemia; one or two diseases? *Brit. J. Haematol.*, **42**, 493-498.

CHAMPLIN, R.E. & GALE, R.P. (1991). *New Strategies in Bone Marrow Transplantation*. Wiley-Liss: New York.

CHAUDHARY, P.M. & RONINSON, I.B. (1991). Expression and activity of P-glycoprotein, a multidrug efflux pump, in human hematopoietic stem cells. *Cell*, **66**, 85-94.

CHEN, W., PEACE, D.J., ROVIRA, D.K., YOU, S.-G. & CHEEVER, M.A. 1992). T-cell immunity to the joining region of $p210^{BCR-ABL}$ protein. *Proc. Natl. Acad. Sci. USA*, **89**, 1468-1472.

CHESSELLS, J.M., HARDISTY, R.M., RAPSON, N.T. & GREAVES, M.F. (1977). Acute lymphoblastic leukaemia in children: classification and prognosis. *Lancet*, **ii**, 1307-1309.

CLARKE, A.G. & MACLENNAN, K.A. (1986). The many facets of thymic involution. *Immunol. Today*, **7**, 204-205.

CLARKSON, B., BERMAN, E., LITTLE, C., ANDREEFF, M., KEMPIN, S., KOLITZ, J., GABRILOVE, J., ARLIN, Z., MERTELSMANN, R., CUNNINGHAM, I., CASTRO-MALASPINA, H., GULATI, S., O'REILLY, R. & GEE, T. (1990). Update on clinical trials of chemotherapy and bone marrow transplantation in acute myelogenous leukemia in adults at Memorial Sloan-Kettering Cancer Center (MSKCC) 1966 to 1989. In *Acute Myelogenous Leukemia; Progress and Controversies*, Gale, R.P. (ed) pp. 239-272. Wiley-Liss: New York.

CRAIG, J.M., HAWKINS, J.M., YAMADA, T., GANESHAGURU, K., MEHTA, A.B. & SECKER-WALKER, L.M. (1990). First intron and M-bcr breakpoints are restricted to the lymphoid lineage in Philadelphia positive acute lymphoblastic leukemia. *Leukemia*, **4**, 678-681.

CRIST, W.M., & KUN, L.E. (1991). Common solid tumors of childhood. *New Engl. J. Med.*, **324**, 461-471.

CRIST, W.M., PULLEN, D.J., FALLETTA, J. VAN EYS, J., BOROWITZ, M., JACKSON, J., DOWELL, B., FRANKEL, L., QUDDUS, F., RAGAB, A. & VIETTI, T. (1986). Clinical and biological features predict a poor prognosis in acute lymphoid leukemias in infants: a pediatric oncology group study. *Blood*, **67**, 135-140.

DALEY, G.Q. & BALTIMORE, D. (1988). Transformation of an interleukin 3-dependent hematopoietic cell line by the chronic myelogenous leukemia-specific $P210^{bcr/abl}$ protein. *Proc. Natl Acad. Sci. USA.*, **85**, 9312-9316.

DOW, L.W., MARTIN, P., MOOHR, J., GREENBERG, M., MACDOUGALL, L.G., NAJFELD, V. & FIALKOW, P.J. (1985). Evidence for clonal development of childhood acute lymphoblastic leukemia. *Blood*, **66**, 902-907.

DOW, L.W., TACHIBANA, N., RAIMONDI, S.C., LAUER, S.J., WITTE, O.N. & CLARK, S.S. (1989). Comparative biochemical and cytogenetic studies of childhood acute lymphoblastic leukemia with the Philadelphia chromosome and other 22q11 variants. *Blood*, **73**, 1291-1297.

DRAPER, G.J., VINCENT, T.J., O'CONNOR, C.M. & STILLER, C.A. (1991). Socio-economic factors and variation in incidence rates between county districts. In *The Geographical Epidemiology of Childhood Leukaemia and Non-Hodgkin's Lymphoma in Great Britain 1966–83*, Draper, G. (ed) pp. 37–46. OPCS: London.

EASTMAN, A. (1990). Activation of programmed cell death by anticancer agents: cisplatin as a model system. *Cancer Cells*, **2**, 275–280.

EINHORN, L.H. (1990). Treatment of testicular cancer: a new and improved model. *J. Clin. Oncol.*, **8**, 1777–1781.

FARBER, S., TOCH, R., SEARS, E.M. & PINKEL, D. (1956). Advances in chemotherapy of cancer in man. *Adv. Cancer Res.*, **4**, 1–71.

FELIX, C.A., NAU, M.M., TAKAHASHI, T., MITSUDOMI, T., CHIBA, I., POPLACK, D.G., REAMAN, G.H., COLE, D.W., LETTERIO, J.J., WHANG-PENG, J., KNUTSEN, T. & MINNA, J.D. (1992). Hereditary and acquired p53 gene mutations in childhood acute lymphoblastic leukemia. *J. Clin. Invest.*, **89**, 640–647.

FENAUX, P., CASTAIGNE, S., CHOMIENNE, C., DOMBRET, H. & DEGOS, L. (1992). All trans retinoic acid treatment for patients with acute promyelocytic leukemia. *Leukemia*, **6(suppl 1)**, 64–66.

FIALKOW, P.J. (1980). Clonal and stem cell origin of blood cell neoplasms. In *Contemporary Hematology/Oncology*, LoBue J. (ed) pp. 1–46. Plenum: New York.

FIALKOW, P.J. (1984). Clonal evolution of human myeloid leukaemias. In *Genes and Cancer*, Biship, J.M., Rowley, J.D. and Greaves, M.F. (eds) pp. 215–226. Alan R. Liss: New York.

FIALKOW, P.J., SINGER, J.W., RASKIND, W.H., ADAMSON, J.W., JACOBSON, R.J., BERNSTEIN, I.D., DOW, L.W., NAJFELD, V. & VEITH, R. (1987). Clonal development, stem-cell differentiation, and clinical remissions in acute nonlymphocytic leukemia. *N. Engl. J. Med.*, **317**, 468–473.

FORD, A.M., MOLGAARD, H.V., GREAVES, M.F. & GOULD, H.J. (1983). Immunoglobulin gene organisation and expression in haemopietic stem cell leukaemia. *EMBO J.*, **2**, 997–1001.

FUSCOE, J.C., ZIMMERMAN, L.J., LIPPERT, M.J., NICKLAS, J.A., O'NEILL, J.P. & ALBERTINI, R.J. (1991). V(D)J recombinase-like activity mediates *hprt* gene deletion in human fetal T-lymphocytes. *Cancer Res.*, **51**, 6001–6005.

GALE, R.P. & HOELZER, D. (1990). *Acute Lymphoblastic Leukemia*. Wiley Liss: New York.

GALE, R.P. (1990). *Acute Myelogenous Leukemia: Progress and Controversies*. Wiley-Liss: New York.

GARDEMBAS-PAIN, M., FLANDRIN, G., DANIEL, M.-T., LEVERGER, G., BERGER, R. & SCHAISON, G. (1991). Acute myeloid leukemia in children less than two years old: clinical, cytologic and cytogenetic correlations. *Leuk. Lymphoma*, **3**, 365–373.

GEHAN, E.A., SMITH, T.L., FREIREICH, E.J., BODEY, G., RODRIGUEZ, V., SPEER, J. & MCCREDIE, K. (1976). Prognostic factors in acute leukemia. *Sem. Oncol.*, **3**, 271–282.

GIBBONS, B., KATZ, F.E., GANLY, P. & CHESSELLS, J.M. (1990). Infant acute lymphoblastic leukaemia with t(11;19). *Brit. J. Haematol.*, 74, 264–269.

GOLDSCHNEIDER, I., METCALF, D., BATTYE, F. & MANDEL, T. (1979). Analysis of rat hemopoietic cells on the fluorescence-activated cell sorter. I. Isolation of pluripotent hemopoietic stem cells and granulocyte-macrophage progenitor cells. *J. Exp. Med.*, **152**, 419–428.

GRAF, T. (1988). Leukemia as a multistep process: studies with avian retroviruses containing two oncogenes. *Leukemia*, **2**, 127–131.

GREAVES, M.F. & ALEXANDER, F.E. (1992). An infectious etiology for common acute lymphoblastic leukemia in childhood? *Leukemia* (in press).

GREAVES, M.F. (1982). 'Target' cells, differentiation and clonal evolution in chronic granulocytic leukaemia: a 'model' for understanding the biology of malignancy. In *Chronic Granulocytic Leukaemia*, Shaw, M.T. (ed) pp. 15–47. Praeger: Eastbourne.

GREAVES, M.F. (1986). Differentiation-linked leukaemogenesis in lymphocytes. *Science*, **234**, 697–704.

GREAVES, M.F. (1988). Speculations on the cause of childhood acute lymphoblastic leukemia. *Leukemia*, **2**, 120–125.

GREAVES, M.F., CHAN, L.C., FORD, A.M., PEGRAM, S.M. & WIEDEMANN, L.M. (1991). Etiological mechanisms in childhood acute lymphoblastic leukemia. In *Childhood Leukemia: Present Problems and Future Prospects*. Kobayashi, N., Akera, T., Mizutani, S. (eds) pp. 3–22. Kluwer: London.

GREAVES, M.F., JANOSSY, G., PETO, J. & KAY, H. (1981). Immunologically defined subclasses of acute lymphoblastic leukaemia in children: their relationship to presentation features and prognosis. *Brit. J. Haematol.*, **48**, 179–197.

GREAVES, M.F., PEGRAM, S.M. & CHAN, L.C. (1985). Collaborative group study of the epidemiology of acute lymphoblastic leukaemia subtypes: background and first report. *Leuk. Res.*, **9**, 715–733.

GREAVES, M.F., SIEFF, C. & EDWARDS, P.A.W. (1983). Monoclonal antiglycophorin as a probe for erythroleukaemias. *Blood*, **61**, 645–651.

GREAVES, M.F., VERBI, W., REEVES, B.R., HOFFBRAND, A.V., DRYSDALE, H.C., JONES, L., SACKER, L.S. & SAMARATUNGA, I. (1979). 'Pre-B' phenotypes in blast crisis of Ph1 positive CML: evidence for a pluripotential stem cell 'target'. *Leuk. Res.*, **3**, 181–191.

GRIFFITHS, S.D., GOODHEAD, D., WRIGHT, E. & GREAVES, M.F. Ultra-sensitivity of B cell precursors to ionizing radiation and apoptosis inducing drugs. (Manuscript in preparation).

HABER, D.A. & HOUSMAN, D.E. (1991). Rate-limiting steps: the genetics of pediatric cancers. *Cell*, **64**, 5–8.

HALL, P.A. & WATT, F.M. (1989). Stem cells: the generation and maintenance of cellular diversity. *Development*, **106**, 619–633.

HARDY, J.R., WILTSHAW, E., BLAKE, P.R., HARPER, P., SLEVIN, M., PERREN, T.J. & TAN, S. (1991*b*). Cisplatin and carboplatin in combination for the treatment of stage IV ovarian carcinoma. *Ann. Oncol.*, **2**, 131–136.

HARDY, R.R., CARMACK, C.E., SHINTON, S.A., KEMP, J.D. & HAYAKAWA, K. (1991*a*). Resolution and characterization of pro-B and pre-pro-B cell stages in normal mouse bone marrow. *J. Exp. Med.*, **173**, 1213–1225.

HENDERSON, E.S., HOELZER, D. & FREEMAN, A.I. (1990). The treatment of acute lymphoblastic leukemia. In *Leukemia*, Henderson, E.S. & Lister, T.A. (eds) pp. 443–484. W.B. Saunders Publ.: Philadelphia.

HERNANDEZ, A., OSTERHOLZ, J., PRICE, C.M., WIEDEMANN, L.M., GORDON, M.Y., GOLDMAN, J.M. & MORGAN, G.J. (1990). Detection of the hybrid BCR/ABL messenger RNA in single CFU-GM colonies using the polymerase chain reaction. *Exp. Hematol.*, **18**, 1142–1144.

HINCHLIFFE, J.R. (1981). Cell death in embryogenesis. In *Cell Death in Biology and Pathology*, Bowen, I.D. & Locksin, R.A. (eds) pp. 35–78. Chapman and Hall: London.

HIRSCH-GINSBERG, C., CHILDS, C., CHANG, K.-S., BERAN, M., CORK, A., REUBEN, J., FREIREICH, E.J., CHANG., L.C.M., BOLLUM, F.J., TRUJILLO, J. & STASS, S.A. (1988). Phenotypic and molecular heterogeneity in Philadelphia chromosome-positive acute leukemia. *Blood*, **71**, 186–195.

HOCKENBERY, D., NUÑEZ, G., MILLIMAN, C., SCHREIBER, R.D. & KORSMEYER, S.J. (1990). Bcl-2 is an inner mitochondrial membrane protein that blocks programmed cell death. *Nature*, **348**, 334.

JANOSSY, G., WOODRUFF, R.K., PAXTON, A., GREAVES, M.F., CAPELLARO, D., KIRK, B., INNES, E.M., EDEN, O.B., LEWIS, C., CATOVSKY, D. & HOFFBRAND, A.V. (1978). Membrane marker and cell separation studies in Ph[1]-positive leukemia. *Blood*, **51**, 861–875.

JANOSSY, G., WOODRUFF, R.K., PIPPARD, M.J., PRENTICE, G., HOFFBRAND, A.V., PAXTON, A., LISTER, T.A., BUNCH, C. & GREAVES, M.F. (1979). Relation of 'lymphoid' phenotype and response to chemotherapy incorporating vincristine-prednisolone in the acute phase of Ph1 positive leukaemia. *Cancer*, **43**, 426–434.

JUNG, S. & SCHLUESENER, H.J. (1991). Human T lymphocytes recognize a peptide of single point-mutated, oncogenic ras proteins. *J. Exp. Med.*, **173**, 273–276.

KALWINSKY, D., MIRRO, J. & DAHL, G.V. (1988). Biology and therapy of childhood acute nonlymphocytic leukemia. *Pediatr. Ann.*, **17**, 172–190.

KASTAN, M.B., SCHLAFFER, E., RUSSO, J.E., COLVIN, O.M., CIVIN, C.I. & HILTON, J. (1990). Direct demonstration of elevated aldehyde dehydrogenase in human hematopoietic stem cells. *Blood*, **75**, 1947–1950.

KINCADE. P.W. (1987). Experimental models for understanding B lymphocyte formation. *Adv. Immuno.*, **41**, 181–267.

KINLEN, L.J., CLARKE, K. & HUDSON, C. (1990). Evidence from population mixing in British New towns 1946–85 on an infective basis for childhood leukaemia. *Lancet*, **336**, 577–582.

KITANO, K., SATO, Y., SUDA, T. & MIURA, Y. (1988). Difference of cell lineage expression of haematopoietic progenitor cells in Philadelphia-positive acute lymphoblastic leukaemia and chronic myelogenous leukaemia. *Brit. J. Haematol.*, **70**, 21–26.

KNUDSON, A.G. (1977). Genetics and etiology of human cancer. *Adv. Hum. Genet.*, **8**, 1–66.

KUNKEL, T.A., GOPINATHAN, K.P., DUBE, D.K., SNOW, E.T. & LOEB, L.A. (1986). Rearrangements of DNA mediated by terminal transferase. *Proc. Natl Acad. Sci. USA*, **83**, 1867–1871.

LAJTHA, L.G. (1979). Stem cell concepts. *Differentiation*, **14**, 23–34.

LANE, D.P. (1992). p53, guardian of the genome. *Nature*, **368**, 15–16.

LE DOUARIN, N.M. (1978). Ontogeny of hematopoietic organs studied in avian embryo interspecific chimeras, in differentiation of normal and neoplastic hematopoietic cells. *Cold Spring Harbor Conf. Cell Proliferation*, **5**, 5–31.

LEARY, A.G., HIRAI, Y., KISHIMOTO, T., CLARK, S.C. & OGAWA, M. (1989). Survival of hemopoietic progenitors in the $G_0$ period of the cell cycle does not require early hemopoietic regulators. *Proc. Natl Acad. Sci. USA*, **86**, 4535–4538.

LIEBER, M.R. (1992). The mechanism of V(D)J recombination: a balance of diversity, specificity, and stability. *Cell*, **70**, 873–876.

LIU, Y.-J., MASON, D.Y., JOHNSON, G.D., ABBOT, S., GREGORY, C.D., HARDIE, D.L., GORDON, J. & MACLENNAN, I.C.M. (1991). Germinal center cells express bcl-2 protein after activation by signals which prevent their entry into apoptosis. *Eur. J. Immunol.*, **21**, 1905–1910.

MAGRATH, I.T. (1990). *The Non-Hodgkin's Lymphomas*. Edward Arnold: London.

MARKS, S.M., BALTIMORE, D., MCCAFFREY, R. (1978). Terminal transferase as a predictor of initial responsiveness to vincristine and prednisone in blastic chronic myelogenous leukemia. *N. Engl. J. Med.*, **298**, 812–814.

MARUYAMA, Y. & FEOLA, J.M. (1987). Relative radiosensitivities of the thymus, spleen, and lymphohemopoietic systems. *Adv. Rad. Biol.*, **12**, 1–82.

MAURER, J., JANSSEN, J.W.G., THIEL, E., VAN DENDEREN, J., LUDWIG, W.-D., AYDEMIR, Ü., HEINZE, B., FONATASCH, C., HARBOTT, J., REITER, A., RIEHM, H., HOELZER, D. & BARTRAM, C.R. (1991). Detection of chimeric BCR-ABL genes in acute lymphoblastic leukaemia by the polymerase chain reaction. *Lancet*, **337**, 1055–1058.

MAYER, P.J., BRADLEY, M.O. & NICHOLS, W.W. (1986). Incomplete rejoining of DNA double-strand breaks in unstimulated normal human lymphocytes. *Mutation Res.*, **166**, 275–285.

MCCULLOCH, E.A., CURTIS, J.E., MESSNER, H.A., SENN, J.S. & GERMANSON, T.P. (1982). The contribution of blast cell properties to outcome variation in acute myeloblastic leukemia. *Blood*, **59**, 601–608.

MCDONNELL, T.J., DEANE, N., PLATT, F.M., NUNEZ, G., JAEGER, U., MCKEARN, J.P. & KORSMEYER, S.J. (1989). *bcl*-2-Immunoglobulin transgenic mice demonstrate extended B cell survival and follicular lymphoproliferation. *Cell*, **57**, 79–88.

MCGUIRE, W.L., GOLDIE, J.H., SALMON, S.E. & LING, V. (1985). Strategies to identify or prevent drug resistance in cancer. *Breast Cancer Res. Treat.*, **5**, 257–268.

MCMANAWAY, M.E., NECKERS, L.M., LOKE, S.L., AL-NASSER, A.A., REDNER, R.L., SHIRAMIZU, B.T., GOLDSCHMIDTS, W.L., HUBER, B.E., BHATIA, K. & MAGRATH, I.T. (1990). Tumour-specific inhibition of lymphoma growth by an antisense oligodeoxynucleotide. *Lancet*, **335**, 808–811.

MEISTRICH, M.L. & VAN BEEL, M.E.A.B. (1990). Radiation sensitivity of the human testis. *Adv. Rad. Biol.*, **14**, 227–268.

METCALF, D. (1985). The granulocyte-macrophage colony-stimulating factors. *Science*, **229**, 16–22.

MEYERS, P.A. (1987). Malignant bone tumors in children: osteosarcoma. In *Hematology/Oncology Clinics of North America: Cancer in Children*, Pochedly, C. (ed) pp. 655–666. W.B. Saunders: Philadelphia.

MILLER, S.C. & OSMOND, D.G. (1975). Lymphocyte populations in mouse bone marrow: quantitative kinetic studies in young, pubertal and adult C3H mice. *Cell Tissue Kinet.*, **8**, 97–110.

NEEFJES, J.J. & PLOEGH, H.L. (1992). Intracellular transport of MHC class II molecules. *Immunol. Today*, **13**, 179–184.

NICHOLS, C.R., ROTH, B.J., HEEREMA, N., GRIEP, J. & TRICOT, G. (1990). Hematologic neoplasia associated with primary mediastinal germ-cell tumors. *New Engl. J. Med.*, **322**, 1425–1429.

PECKHAM, M. (1981). *The Management of Testicular Tumours*. Edward Arnold; London.

PETERSON, L.C., BLOOMFIELD, C.D. & BRUNNING, R.D. (1976). Blast crisis as an initial or terminal manifestation of chronic myeloid leukemia. A study of 28 patients. *Am. J. Med.*, **60**, 209–220.

PIERCE, G.B. & SPEERS, W.C. (1988). Tumors as caricatures of the process of tissue renewal: prospects for therapy by directing differentiation. *Cancer Res.*, **48**, 1996–2004.

PINKEL, D. (1979). The ninth annual David Karnofsky lecture. Treatment of acute lymphocytic leukemia. *Cancer*, **43**, 1128–1137.

PINKEL, D. (1987). Curing children of leukemia. *Cancer*, **59**, 1683–1691.

POCHEDLY, C. (1987). *Hematology/Oncology Clinics of North America: Cancer in Children*. W.B. Saunders: Philadelphia.

POTTEN, C.S. & LOEFFLER, M. (1990). Stem cells: attributes, cycles, spirals, pitfalls and uncertainties. Lessons for and from the Crypt. *Development*, **110**, 1001–1020.

PRICE, C.M., KANFER, E.J., COLMAN, S.M., WESTWOOD, N., BARRETT, A.J. & GREAVES, M.F. (1992). Simultaneous genotypic and immunophenotypic analysis of interphase cells using dual colour fluorescence: a demonstration of lineage involvement in polycythemia vera. *Blood*, **80**, 1033–1038.

PUI, C.-H., CRIST, W.M. & LOOK, A.T. (1990). Biology and clinical significance of cytogenetic abnormalities in childhood acute lymphoblastic leukemia. *Blood*, **76**, 1449–1463.

RAIMONDI, S.C., PEIPER, S.C., KITCHINGMAN, G.R., BEHM, F.G., WILLIAMS, D.L., HANCOCK, M.L. & MIRRO, J. (1989). Childhood acute lymphoblastic leukemia with chromosomal breakpoints at 11q23. *Blood*, **73**, 1627–1634.

REED, J.C., STEIN, C., SUBASINGHE, C., HALDAR, S., CROCE, C.M., YUM, S. & COHEN, J. (1990). Antisense-mediated inhibition of *BCL2* protooncogene expression and leukemic cell growth and survival: comparisons of phosphodiester and phosphorothioate oligodeoxynucleotides. *Cancer Res.*, **50**, 6565–6570.

RIBEIRO, R.C., ABROMOWITCH, M., RAIDMONDI, S.C., MURPHY, S.B., BEHM, F. & WILLIAMS, D.L. (1987). Clinical and biologic hallmarks of the Philadelphia chromosome in childhood acute lymphoblastic leukemia. *Blood*, **70**, 948–953.

REIDER, H., FONATSCH, C. & FREUND, M. (1991). Abnormalities of the short arm chromosome 9. A nonrandom secondary aberration in Philadelphia chromosome-positive acute lymphoblastic leukemia (ALL). *Cancer Genet. Cytogenet.*, **53**, 139–142.

RIEHM, H. (1991). Results and significance of six randomized trials in four consecutive ALL-BEM studies. In *Childhood Leukemia: Present Problems and Future Prospects*, Kobayashi, N., Akera, T., Mizutani, S. (eds) pp. 135–147. Kluwer: London.

ROHATINER, A.Z.S. & LISTER, T.A. (1990). The treatment of acute myelogenous leukaemia. In *Leukaemia*, Henderson, E.S., Lister, T.A. (eds) pp. 485. W.B. Saunders: Philadelphia.

ROHATINER, A.Z.S., DAVIS, C.L., LIM, J., AMESS, J. & LISTER, T.A. (1990). Survival with acute myelogenous leukaemia. In *Acute Myelogenous Leukemia: Progress and Controversies*, Gale, R.P. (ed) pp. 323–331. Wiley-Liss: New York.

ROLINK, A. & MELCHERS, F. (1991). Molecular and cellular origins of B lymphocyte diversity. *Cell*, **66**, 1081–1094.

ROTH, B.J. & NICHOLS, C.R. (1992). *Seminars in Oncology: Testicular Cancer*. Vol 19.

RUSSO, C., CARROLL, A., KOHLER, S., BOROWITZ, M., AMYLON, M., HOMANS, A., KEDAR, A., SHUSTER, J., LAND, V., CRIST, W., PULLEN, J. & LINK, M. (1991). Philadelphia chromosome and monosomy 7 in childhood acute lymphoblastic leukemia: a pediatric oncology group study. *Blood*, **77**, 1050–1056.

SALLAN, S.E., RITZ, J., PESANDO, J., GELBER, R., O'BRIEN, C., HITCHCOCK, S., CORAL, F. & SCHLOSSMAN, S.F. (1980). Cell surface antigens: prognostic implications in childhood acute lymphoblastic leukemia. *Blood*, **55**, 395–402.

SCHATZ, D.G., OETTINGER, M.A. & SCHLISSEL, M.S. (1992). V(D)J recombination: molecular biology and regulation. *Ann. Rev. Immunol.*, **10**, 359–384.

SCHELLONG, G., CREUTZIG, U. & RITTER, J. (1990). Improved survival of children with acute myelogenous leukemia (AML): update of the studies AML BFM-78 and BFM-83. In *Acute Myelogenous Leukemia: Progress and Controversies*, Gale, R.P. (ed) pp. 165–174. Wiley-Liss: New York.

SECKER-WALKER, L.M. & CRAIG, J.M. (1993). Prognostic implications of breakpoint and lineage heterogeneity in Philadelphia-positive acute lymphoblastic leukemia: a review. *Leukemia* (in press).

SECKER-WALKER, L.M., CRAIG, J.M., HAWKINS, J.M. & HOFFBRAND, A.V. (1991). Philadelphia positive acute lymphoblastic leukemia in adults: age distribution, BCR breakpoint and prognostic significance. *Leukemia*, **5**, 196–199.

SELLINS, K.S. & COHEN, J.J. (1987). Gene induction by γ-irradiation leads to DNA fragmentation in lymphocytes. *J. Immunol.*, **139**, 3199–3206.

SHAW, M.T. (1982). *Chronic Granulocytic Leukaemia*. Praeger: Eastbourne.

SIMONE, J.V. (1979*a*). Childhood leukemia as a model for cancer research: the Richard and Hinda Rosenthal Foundation Award Lecture. *Cancer Res.*, **39**, 4301–4307.

SIMONE, J.V. (1979*b*). Childhood acute lymphocytic leukemia – a model for therapeutic strategies in hemopoietic neoplasia. In *Recent Results in Cancer Research – Strategies in Clinical Haematology*, Gross, R. & Hellriegel, K.P. (eds) pp. 49–54. Springer-Verlag: Berlin.

SKIPPER, H.E. & SCHABEL, F.M. (1984). Tumor stem cell heterogeneity: implications with respect to classification of cancers by therapeutic effect. *Cancer Treat. Rep.*, **68**, 43–62.

SPOONCER, E., HEYWORTH, C.M., DUNN, A. & DEXTER, T.M. (1986). Self-renewal and differentiation of interleukin-3-dependent multipotent stem cells are modulated by stromal cells and serum factors. *Differentiation*, **31**, 111–118.

STEIN, W.D. (1991). Analysis of cancer incidence data on the basis of multistage and clonal growth models. *Adv. Cancer Res.*, **56**, 161–213.

STONG, R.C., KORSMEYER, S.J., PARKIN, J.L., ARTHUR, D.C. & KERSEY, J.H. (1985). Human acute leukemia cell line with the t(4;11) chromosomal rearrangement exhibits B lineage and monocytic characteristics. *Blood*, **65**, 21–31.

STRASSER, A., ROLINK, A. & MELCHERS, F. (1989). One synchronous wave of B cell development in mouse fetal liver changes at day 16 of gestation from dependence to independence of a stromal cell environment. *J. Exp. Med.*, **170**, 1973–1986.

SZCZYLIK, C., SKORSKI, T., NICOLAIDES, N.C., MANZELLA, L., MALAGUARNERA, L., VENTURELLI, D., GEWIRTZ, A.M. & CALABRETTA, B. (1991). Selective inhibition of leukemia cell proliferation by BCR-ABL antisense oligodeoxynucleotides. *Science*, **253**, 562–565.

TACHIBANA, N., RAIMONDI, S.C., LAUER, S.J., SARTAIN, P. & DOW, L.W. (1987). Evidence for a multipotential stem cell disease in some childhood Philadelphia chromosome positive acute lymphoblastic leukemia. *Blood*, **70**, 1458–1461.

TROTT, K.-R. (1984). The cellular interpretation of tumor radioresistance. *Cancer Treat. Rev.*, **11 (suppl A)**, 81–83.

TROWELL, O.A. (1952). The sensitivity of lymphocytes to ionizing radiation. *J. Pathol.*, **64**, 687.

TURHAN, A.G., EAVES, C.J., KALOUSEK, D.K., EAVES, A.C. & HUMPHRIES, R.K. (1988). Molecular analysis of clonality and bcr rearrangements in Philadelphia chromosome-positive acute lymphoblastic leukemia. *Blood*, **71**, 1495–1498.

VAN HEYNINGEN, V. & HASTIE, N.D. (1992). Wilms' tumour: reconciling genetics and biology. *Trends Genet.*, **85**, 16–21.

VOGELSTEIN, B., FEARON, E.R., HAMILTON, S.R.,. KERN, S.E., PREISINGER, A.C., LEPPERT, M., NAKAMURA, Y., WHITE, R., SMITS, A.M.M. & BOS, J.L. (1988). Genetic alterations during colorectal-tumor development. *N. Engl. J. Med.*, **319**, 525–532.

WALDMANN, T.A. (1992). Immune receptors: targets for therapy of leukemia/lymphoma, autoimmune diseases and for the prevention of allograft rejection. *Ann. Rev. Immunol.*, **10**, 675–704.

WEISS, S. & BOGEN, B. (1991). MHC class II-restricted presentation of intracellular antigen. *Cell*, **64**, 767–776.

WHITTEMORE, A.S. (1978). Quantitative theories of oncogenesis. *Adv. Cancer Res.*, **27**, 55–88.

WYLLIE, A.H. (1981). Cell death: a new classification separating apoptosis from necrosis. In *Cell Death in Biology and Pathology*, Bowen, I.D. & Locksin, R.A. (eds) pp.9–34. Chapman and Hall: London.

YAMADA, M., WASSERMAN, R., LANGE, B., REICHARD, B.A., WOMER, R.B. & ROVERA, G. (1990). Minimal residual disease in childhood B-lineage lymphoblastic leukemia. *N. Eng. J. Med.*, **323**, 448–455.

YOKOTA, S., HANSEN-HAGGE, T.E., LUDWIG, W.-D., REITER, A., RAGHAVACHAR, A., KLEIHAUER, E. & BARTRAM, C.R. (1991). Use of polymerase chain reactions to monitor minimal residual disease in acute lymphoblastic leukemia patients. *Blood*, **77**, 331–339.